# Figure-eight thermal hysteresis of aminomethylenehelicene oligomers with terminal C_16_ alkyl groups during hetero-double-helix formation†

**DOI:** 10.1039/c9sc06496f

**Published:** 2020-02-26

**Authors:** Tsukasa Sawato, Rina Iwamoto, Masahiko Yamaguchi

**Affiliations:** Department of Organic Chemistry, Graduate School of Pharmaceutical Sciences, Tohoku University Aoba Sendai 980-8578 Japan yama@m.tohoku.ac.jp +81 22-795-6811

## Abstract

1 : 1 mixtures of aminomethylenehelicene (*P*)-tetramer and (*M*)-pentamer with terminal C_16_ alkyl groups in fluorobenzene showed structural changes between hetero-double-helices **B** and **C** and random-coils 2**A**. Figure-eight thermal hysteresis appeared when the solution was cooled and heated at a constant rate and involved the crossing of cooling and heating curves in Δ*ε*/temperature profiles. This unusual thermal hysteresis emerged in the intermediate state between counterclockwise and clockwise thermal hystereses. This phenomenon arose from the competition between self-catalytic reactions to form **B** and **C** from 2**A**. Significant effects of terminal C_16_ alkyl groups on the thermodynamic and kinetic phenomena are also described.

## Abstract 2

1 : 1 mixtures of aminomethylenehelicene (*P*)-tetramer and (*M*)-pentamer with terminal C_16_ alkyl groups in fluorobenzene showed structural changes between hetero-double-helices **B** and **C** and random-coils 2**A**.

## Graphical abstract

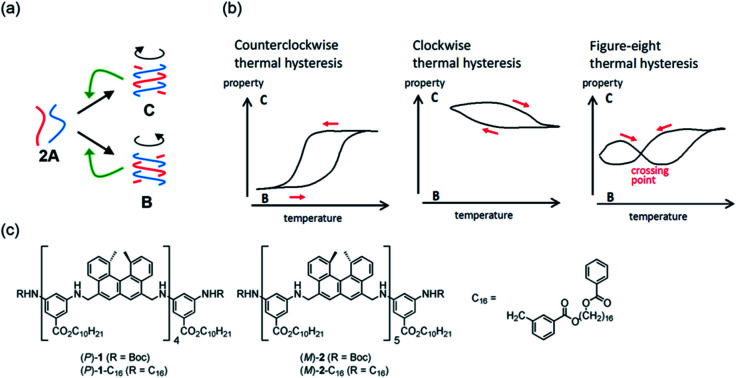

## Introduction

Hysteresis is the dependence of a state on its history and, alternatively, the time delay between an input and an output, and it appears broadly in nature, materials, environments, biological phenomena, and societies.^1,2^ Magnetic hysteresis is derived from external magnetic field strength (input) and magnetization (output).^3^ Once magnetized, a magnet remains magnetized indefinitely, and even when the field is removed, the alignment of magnetic particles remains. Ferroelectric materials show hysteresis in spontaneous electric polarization (output) that can be reversed by the application of an external electric field (input).^4^ Electrical hysteresis appears in electrical current (output) in response to changes in voltage (input).^5^ Phase transition from a liquid to a solid shows thermal hysteresis, as seen in supercooling, in which a temperature change (input) induces structural changes (output) with a time delay.^6^ The amounts of adsorbed and desorbed gas (output) on solid materials exhibit hysteresis during changes in pressure (input).^7^ Regarding sediment pollution, water-quality degradation, and ecosystem impairment, the suspended-sediment concentration (output) in a river shows hysteresis in response to flow (input).^8^ Traffic exhibits hysteresis with regard to flow (input) and occupancy (output) of cars in a city.^9^ Hysteresis also occurs in biological phenomena, which are based on reversible chemical reactions expressed by the concentration of a product (output) in response to changes in intensity of external stimulations (input), which include substrate/ligand/catalyst concentration, temperature, and pH.^10,11^ Hysteresis is experimentally expressed using profiles of input and output properties, which provide different output loops in response to increases and decreases in input intensity. It is therefore interesting to develop hysteresis materials, to explore and understand the physical implications of their properties and to apply such materials in devices.

Hysteresis is a complex phenomenon. Physics-based models and phenomenological models have been suggested, and the classification of hysteretic phenomena has been attempted: (1) rate-dependent hysteresis is defined by the disappearance of hysteresis with a decreasing input rate, whereas rate-independent hysteresis is not affected by the input rate.^12,13^ (2) The shapes of hysteresis loops are classified into six types: leaf, crescent, classical, tilted classical, double loop, and bat.^14^ (3) Four types of loops of hysteresis curves are described by the IUPAC in the adsorption and desorption of gases as a function of pressure.^15^ (4) We propose symmetric and asymmetric types of hysteresis, both of which result from the nature of the input. Magnetic and electrical hystereses are symmetric because their inputs differ only in direction but are identical in intensity; thermal and biological hystereses are asymmetric because their input of high and low temperatures and concentrations differ in intensity.

Among the various types of hystereses, figure-eight or pinched hysteresis is of interest, in which the loops in the input–output profiles cross each other. Electrical pinched hysteresis has attracted much interest with regard to the development of memristors,^16–22^ which are considered to be the fourth basic circuit element. Figure-eight hysteresis appears in magnetic,^23^ thermal,^24,25^ mechanical,^26^ sediment,^27–29^ traffic,^30^ and medical phenomena.^31^ It is therefore interesting to study figure-eight hysteresis in chemical reactions, which, however, has not been examined yet.

Chemical reactions are involved in diverse natural and biological phenomena, and their clarification and control are critical. A chemical reaction is described by the concepts of quantum and statistical mechanics.^32–34^ In quantum mechanics, the structural changes of molecules and their relative thermodynamic stability involving diverse microscopic pathways, called microscopic mechanisms, are analyzed. In statistical mechanics, the flux or number of molecules that visit the pathways, called macroscopic mechanisms, is analyzed. Such chemical reactions can involve rearrangement of covalent and noncovalent bonds.

We have been studying the thermal hysteresis of noncovalent chemical reactions considering both microscopic and macroscopic mechanisms.^32–34^ We have reported that thermal hysteresis can appear in reversible noncovalent chemical reactions of molecules dispersed in solution, which involve different macroscopic mechanisms during cooling and heating. This phenomenon is experimentally shown using different cooling and heating curves in profiles, in which cooling and heating as the input provide substrate/product properties and concentrations as the output. Accordingly, thermal hysteresis in a noncovalent chemical reaction of molecules dispersed in solution can be analyzed with regard to substrate/product concentrations. The system is closed, and the experiments do not involve the addition or removal of matter, which eliminates the use of a large number of factors in the analysis of hysteresis. Thermal hysteresis is asymmetric and rate-dependent and is sensitive to subtle changes in the environment, including the rate of temperature change and thermal history.

**Fig. 1:**
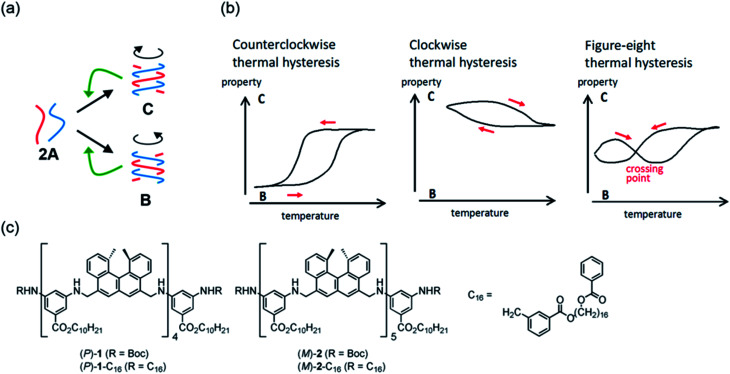
(a) Chemical system involving competitive self-catalytic reactions to form **B** and **C** from 2**A**. (b) Counterclockwise, clockwise, and figure-eight thermal hystereses. (c) Chemical structures of aminomethylenehelicene oligomers.

In this paper, we describe figure-eight thermal hysteresis during a noncovalent chemical reaction of molecules dispersed in solution, in which cooling and heating curves cross each other in Δ*ε*/temperature profiles obtained by circular dichroism (CD) spectroscopy (Fig. 1). Depending on the cooling and heating rate, clockwise and counterclockwise thermal hystereses appeared, and figure-eight thermal hysteresis appeared in the intermediate state (Fig. 1b).^35,36^ Note that Δ*ε* can be related to the concentrations of substrates and products, which provides a concise mechanistic model. This work can be a basis to understand and compare the hysteresis phenomena in different fields.

We previously developed a 1 : 1 mixture of aminomethylenehelicene (*P*)-tetramer (*P*)-**1** and (*M*)-pentamer (*M*)-**2** in solution (Fig. 1c), which formed random-coils 2**A** and hetero-double-helices **B** and **C** due to competition of self-catalytic reactions to form **B** and **C** from 2**A**.^37–39^ It was noted that **B** and **C** possessed enantiomeric structures with right- and left-handed helices. In this work, we synthesized aminomethylenehelicene (*P*)-tetramer (*P*)-**1**-C_16_ and (*M*)-pentamer (*M*)-**2**-C_16_ with C_16_ alkyl terminal groups, which exhibited figure-eight thermal hysteresis. The phenomenon was analyzed with regard to Δ*ε* and concentrations of **A**, **B**, and **C**. It was noted that the thermodynamic and kinetic phenomena were significantly affected by the terminal C_16_ alkyl groups.

## Results and discussion

### Figure-eight thermal hysteresis of (*P*)-**1**-C_16_/(*M*)-**2**

(*P*)-**1**-C_16_ and (*M*)-**2**-C_16_ were synthesized by coupling reactions of (*P*)-**1** and (*M*)-**2**,^37–39^ respectively, with the C_16_ ester of *m*-formylbenzoic acid (Scheme S1†). Three combinations (*P*)-**1**-C_16_/(*M*)-**2**, (*P*)-**1**/(*M*)-**2**-C_16_, and (*P*)-**1**-C_16_/(*M*)-**2**-C_16_ were obtained, and their properties were compared with those of the original (*P*)-**1**/(*M*)-**2**.

**Fig. 2:**
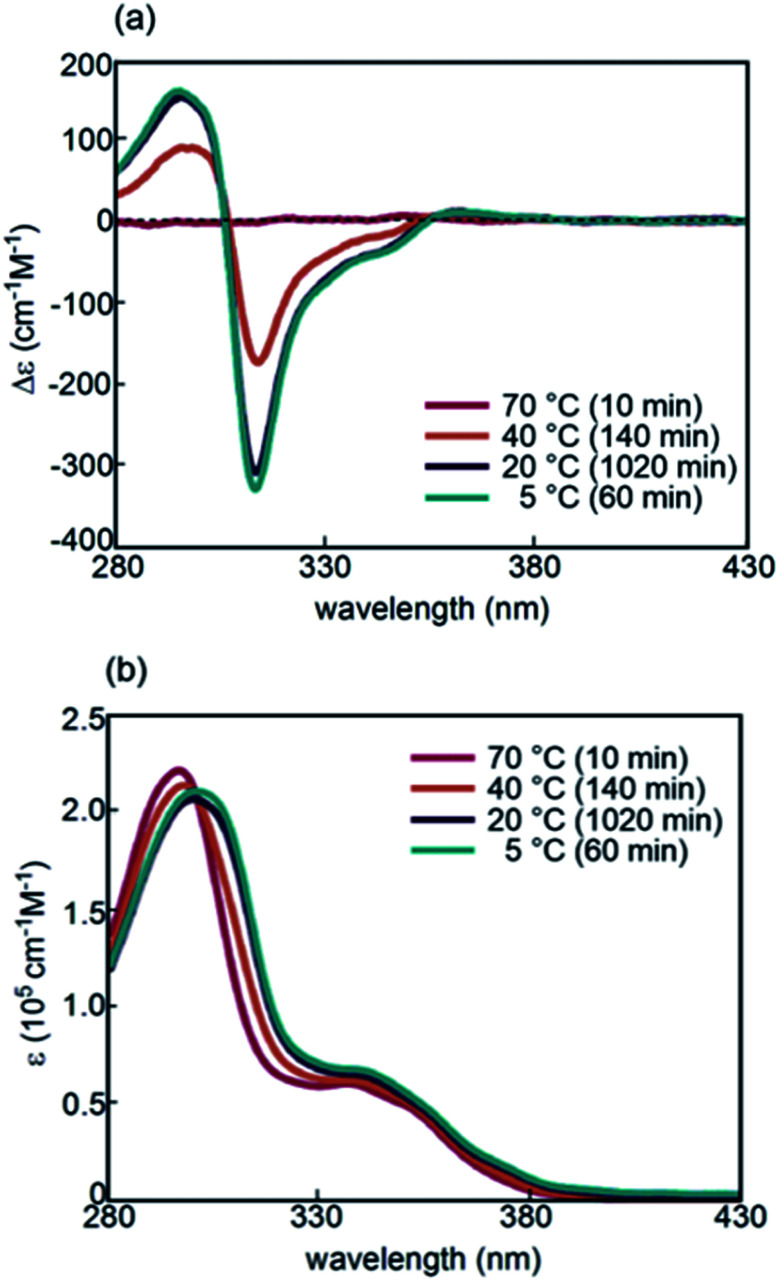
(a) CD spectra and (b) UV-Vis spectra of (*P*)-**1**-C_16_/(*M*)-**2** in fluorobenzene (0.5 mM) at 70, 40, 20, and 5 °C after the time shown in parentheses.

Formation of random-coils 2**A** and hetero-double-helix **B** by (*P*)-**1**-C_16_/(*M*)-**2** was determined in fluorobenzene (0.5 mM) to be analogous to that with the (*P*)-**1** and (*M*)-**2** system.^32–34^ A solution of (*P*)-**1**-C_16_/(*M*)-**2** was heated to 70 °C, and the CD spectra show a very weak Cotton effect of 2**A** (Fig. 2a). Cooling from 70 to 40, 20, and 5 °C resulted in a significant decrease of the Cotton effect at 314 nm and a change in the absorption maximum in the UV-Vis spectra from 296 to 300 nm (Fig. 2b). The spectroscopic analyses at various temperatures and concentrations using different solvents resulted in the equilibrium shifted S-state of **B** with a Δ*ε* of −430 cm^−1^ M^−1^ and an *ε* of 1.6 × 10^5^ cm^−1^ M^−1^ at 314 nm, in which essentially all the molecules are **B** (Fig. S1†). Dynamic light scattering (DLS) analysis showed average diameters of 1 nm for 2**A** and 10 nm for **B**, which indicated that molecules are dispersed in solution without forming larger aggregates.

**Fig. 3:**
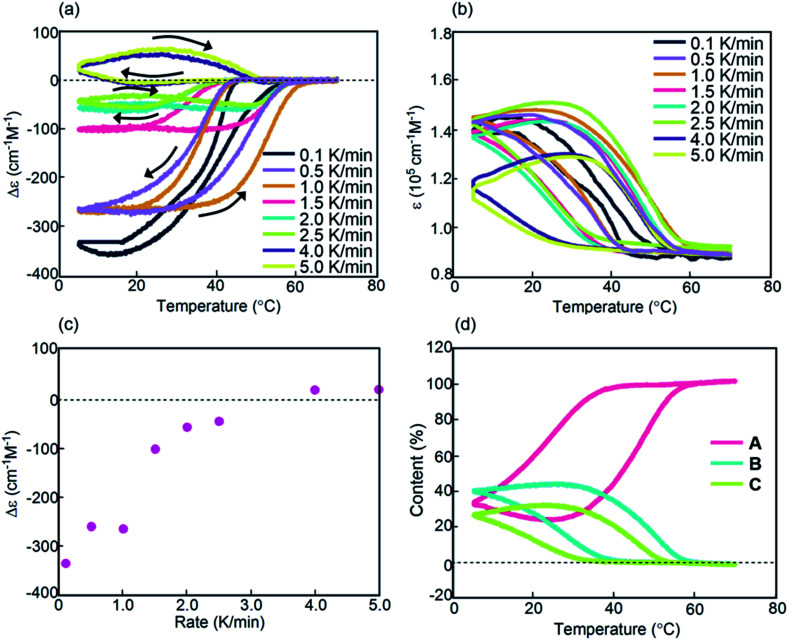
Experiments on cooling and heating at constant rates between 0.1 and 5.0 K min^−1^ using (*P*)-**1**-C_16_/(*M*)-**2** in fluorobenzene (0.5 mM), as shown using (a) Δ*ε*(314 nm)/temperature and (b) *ε*(314 nm)/temperature profiles. Temperature was decreased from 70 to 5 °C and then increased from 5 °C to 70 °C. (c) Profiles of Δ*ε*(314 nm) at 5 °C as a function of cooling rate. (d) Content/temperature profiles at 2.0 K min^−1^.

Experiments on cooling and heating the solution (0.5 mM) were conducted between 70 and 5 °C at constant rates between 0.1 and 5.0 K min^−1^, and the results were shown using Δ*ε*(314 nm)/temperature profiles (Fig. 3a). When the solution was cooled and heated at rates of 0.1 and 1.0 K min^−1^, counterclockwise thermal hysteresis loops were obtained between Δ*ε* values of 0 and −340 cm^−1^ M^−1^. At 1.5 K min^−1^, a smaller loop appeared with a slight crossing of cooling and heating curves at 21 °C. At higher heating and cooling rates of 2.0 and 2.5 K min^−1^, the crossing phenomenon clearly appeared, showing figure-eight thermal hysteresis (also see Fig. 5a, dashed lines). We have been studying thermal hysteresis in noncovalent chemical reactions of molecules dispersed in solution using helicene oligomers, and such a phenomenon was not previously observed.^32–34^ When the rate was increased to 4.0 and 5.0 K min^−1^, clockwise thermal hysteresis loops were obtained in the positive Δ*ε* domain between 0 and +62 cm^−1^ M^−1^.

**Fig. 4:**
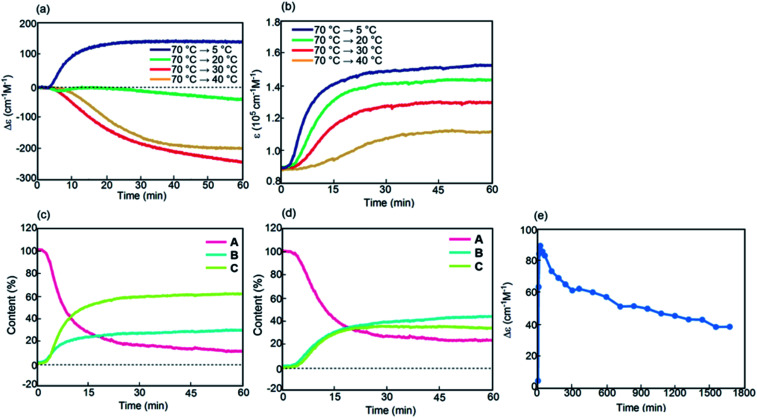
Kinetic analysis under isothermal conditions of (*P*)-**1**-C_16_/(*M*)-**2** in fluorobenzene (0.5 mM) shown using (a) Δ*ε*(314 nm)/time profiles and (b) *ε*(314 nm)/time profiles at 5, 20, 30, and 40 °C for 60 min. Content/time profiles are also shown at (c) 5 °C and (d) 20 °C. (e) A long experiment was conducted at 5 °C as shown using Δ*ε*(314 nm)/time profiles.

**Fig. 5:**
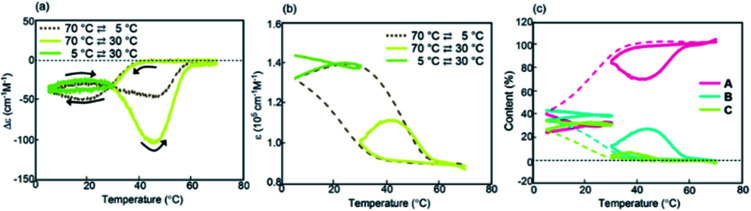
(a) Δ*ε*(314 nm)/temperature profiles and (b) *ε*(314 nm)/temperature profiles of (*P*)-**1**-C_16_/(*M*)-**2** in fluorobenzene (0.5 mM) at a cooling and heating rate of 2.0 K min^−1^. The temperature was decreased from 70 °C to 30 °C and then increased to 70 °C (yellow lines); the temperature was decreased from 70 °C to 5 °C, increased to 30 °C, and then decreased to 5 °C (green lines). (c) Content/temperature profiles are also shown. Dashed lines were obtained from Fig. 3a and d during cooling.

Figure-eight thermal hysteresis appeared at intermediate rates of 1.5 and 2.5 K min^−1^ in the intermediate state of the counterclockwise and clockwise thermal hystereses. It was also noted that figure-eight thermal hysteresis appeared in the domain close to 0 cm^−1^ M^−1^ in the Δ*ε*(314 nm)/temperature profiles. Figure-eight thermal hysteresis was also observed at 0.4 and 0.6 mM concentrations, and the latter provided a loop witha larger area (Fig. S3†).

Values of Δ*ε* at 5 °C at different cooling rates can provide information on the equilibrium state because equilibrium is considered to be a limiting phenomenon for a very slow temperature change. Δ*ε* monotonously decreased with a decrease in the cooling rate (Fig. 3c) and, at slower cooling rates, **B** was formed in higher concentrations, at which point the system was considered to be close to equilibrium.

Thermal hysteresis was also analyzed using *ε*(314 nm)/temperature profiles obtained by UV-Vis analysis at 314 nm (Fig. 3b). At cooling and heating rates between 0.1 and 2.5 K min^−1^, similar thermal hysteresis loops were obtained with a small *ε* value at high temperatures owing to the formation of 2**A** and a large *ε* value at low temperatures owing to the formation of **B**.

It was previously described that thermal hysteresis of (*P*)-**1**/(*M*)-**2** involved the formation of enantiomeric hetero-double-helices,^37–39^ which implied the formation of right- and left-handed hetero-double-helices. It is then reasonable to consider the formation of analogous hetero-double-helices **B** and **C** in (*P*)-**1**-C_16_/(*M*)-**2**. In accordance, the formation of **C** was shown using the inverted CD spectra and the positive Δ*ε* at high cooling and heating rates (Fig. S4d and e†).

The concentrations of **A**, **B**, and **C** can be calculated from Δ*ε* and *ε*, assuming the involvement of three species and identical |Δ*ε*| values for the S-states of **B** and **C** (see the ESI for discussion†). Then, thermal hysteresis was shown using profiles of the contents of **A**, **B**, and **C** as a function of temperature. At a low cooling and heating rate of 0.5 K min^−1^, which provided counterclockwise thermal hysteresis, two-state interconversion between 2**A** and **B** occurred with a minimum formation of **C** (Fig. S2a†). At a high rate of 4.0 K min^−1^, which provided clockwise thermal hysteresis, the formation of **C** dominated that of **B** (Fig. S2b†). At an intermediate rate of 2.0 K min^−1^, which provided figure-eight thermal hysteresis, the formation of **B** competed with that of **C** (Fig. 3d). Analogous to (*P*)-**1**/(*M*)-**2**,^37–39^ involvement of competitive self-catalytic reactions to form **B** and **C** from 2**A** was considered here (Fig. 1a). The downward inflation of Δ*ε*/temperature profiles at 25–45 °C was ascribed to the self-catalytic reaction to form **B** with a negative Δ*ε* at 314 nm, and the upward inflation at 5–20 °C was ascribed to the self-catalytic reaction to form **C** with a positive Δ*ε* (Fig. 3a and 5a). The figure-eight thermal hysteresis is derived from a delicate balance between competitive self-catalytic reactions.

Kinetic analysis under isothermal conditions showed a nonlinear temperature dependence (Fig. 4 and S4†). Solutions at different temperatures were prepared by rapidly cooling solutions from 70 °C to 5, 20, 30, or 40 °C, which initially provided a Δ*ε* of approximately 0 cm^−1^ M^−1^ because of the 2**A** state.

Sigmoidal kinetics with lag times appeared as shown in Δ*ε*/time profiles (Fig. 4a). At 5 °C, Δ*ε* rapidly increased to +140 cm^−1^ M^−1^ in 60 min owing to the formation of **C**. Accordingly, the shape of the CD spectrum was enantiomeric to that of **B** (Fig. S4d†). Content/time profiles were obtained from *ε*(314 nm)/time profiles (Fig. 4b), which showed the formation of **B** and **C**. A long experiment of up to 1680 min showed a very slow decrease in Δ*ε* (Fig. 4e and S4e†), owing to the slow conversion of **C** to **B**. At a higher concentration of 1.0 mM at 5 °C, Δ*ε* remained approximately 0 cm^−1^ M^−1^, which was smaller than that at 0.5 mM (Fig. S6†).

A different phenomenon appeared at 20 °C, and Δ*ε* slowly decreased from 0 cm^−1^ M^−1^ to negative values reaching −40 cm^−1^ M^−1^ after 60 min and still continued to decrease thereafter (Fig. 4a). Because *ε*(314 nm)/time profiles indicated a steady state after 30 min (Fig. 4b), the slow decrease of Δ*ε* was ascribed to the conversion of **C** to **B** (Fig. 4d). At 30 °C, Δ*ε* decreased reaching −240 cm^−1^ M^−1^ after 60 min and still continued to decrease thereafter (Fig. S4b†). At 40 °C, Δ*ε* reached −200 cm^−1^ M^−1^ after 60 min (Fig. 4a). The tendency to form **B** with a negative Δ*ε* indicated higher thermodynamic stability of **B** than of **C**. This behavior is in contrast to that of the previously described (*P*)-**1**/(*M*)-**2**, in which **C** was more stable than **B**.^37–39^ The introduction of the terminal C_16_ groups reversed the relative thermodynamic stability of **B** and **C**.

The properties of (*P*)-**1**-C_16_/(*M*)-**2** were examined at the crossing point of 30 °C under 2.0 K min^−1^ conditions, which showed another interesting feature of the figure-eight thermal hysteresis. When a solution was cooled from 70 to 30 °C and heated to 70 °C at a rate of 2.0 K min^−1^, downward inflation appeared during heating (Fig. 5a, yellow lines). Compared with heating from 5 to 70 °C (Fig. 5a, dashed lines), the downward inflation was significant in the heating from 30 to 70 °C. These results indicate that the reactivity of (*P*)-**1**-C_16_/(*M*)-**2** differs depending on its thermal history to reach the crossing point at 30 °C either from 70 °C or 5 °C. The temperature change rate of 2.5 K min^−1^ showed a similar trend (Fig. S7c†). The 30 to 70 °C experiment was also analyzed using content/temperature profiles, which showed the involvement of interconversion between 2**A** and **B** with minimum formation of **C** (Fig. 5c).

Another experiment between 5 and 30 °C below the crossing point was conducted. A solution was cooled from 70 °C to 5 °C, heated to 30 °C, and cooled to 5 °C (Fig. 5a, green lines), during which a small clockwise thermal hysteresis appeared analogous to the thermal hysteresis obtained in the cooling and heating experiment between 5 and 70 °C (Fig. 5a, dotted lines). The content/temperature profiles obtained in the 5/30 °C experiment showed that the relative concentrations of 2**A**, **B**, and **C** remained unchanged (Fig. 5c). Considerably different phenomena appeared above and below the crossing point.

Cooling and heating experiments were also conducted between 28 and 38 °C; this range included the crossing point (Fig. S8†). Irrespective of starting from 70 °C or 5 °C, repeated experiments showed a gradual decrease in Δ*ε* and small changes in *ε* (Fig. S8a–d†). The content/temperature profiles indicated convergence in the concentrations of **A** and **B**, which was accompanied by a decrease in the concentration of **C** (Fig. S8e and f†).

(*P*)-**1**-C_16_/(*M*)-**2** showed figure-eight thermal hysteresis in its Δ*ε*(314 nm)/temperature profiles, in which cooling and heating curves crossed. Counterclockwise thermal hysteresis appeared at low cooling and heating rates; clockwise thermal hysteresis appeared at high rates; figure-eight thermal hysteresis appeared at intermediate rates of 1.5–2.5 K min^−1^ (Fig. 3a). The figure-eight thermal hysteresis is a phenomenon in the intermediate state between counterclockwise and clockwise thermal hystereses and results from the competition between self-catalytic reactions to form hetero-double-helices **B** and **C** from 2**A**. It was also noted that **B** is thermodynamically more stable than **C**.

### Thermal hysteresis of (*P*)-**1**/(*M*)-**2**-C_16_

An advantage of studies using helicene oligomers is that the combination of these oligomers can be varied, which enables the tuning of properties and promotes systematic understanding of dynamic phenomena. Another combination of aminomethylenehelicene oligomers (*P*)-**1**/(*M*)-**2**-C_16_ in fluorobenzene (0.5 mM) was examined, the structure of which differed from that of (*P*)-**1**-C_16_/(*M*)-**2** in the position of the terminal C_16_ alkyl groups (Fig. 1c and S9†).

**Fig. 6:**
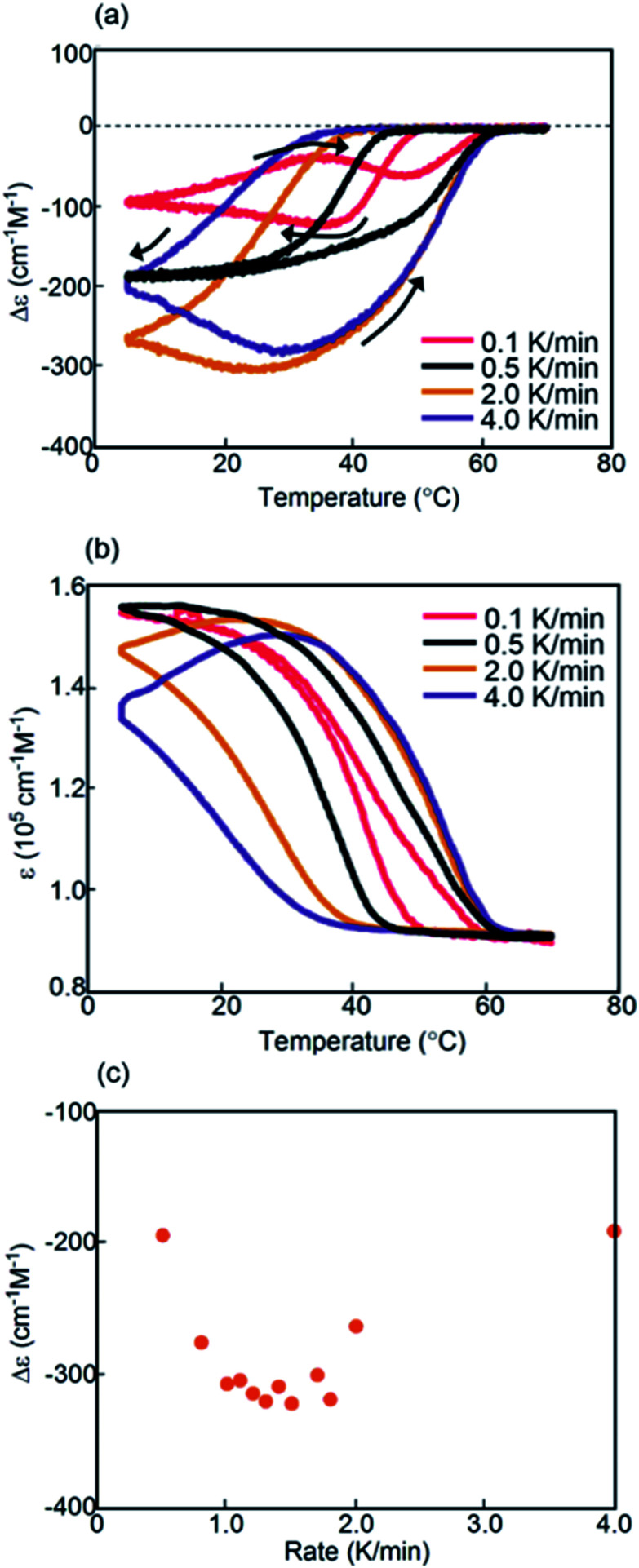
(a) Δ*ε*(314 nm)/temperature profiles and (b) *ε*(314 nm)/temperature profiles of (*P*)-**1**/(*M*)-**2**-C_16_ in fluorobenzene (0.5 mM) at cooling and heating rates of 0.1 to 4.0 K min^−1^. The temperature was decreased from 70 °C to 5 °C and then increased from 5 °C to 70 °C. The data at 0.5 and 2.0 K min^−1^ were obtained from Fig. S10.† (c) Profiles of Δ*ε*(314 nm) at 5 °C as a function of cooling rate.

Constant-rate cooling and heating experiments using (*P*)-**1**/(*M*)-**2**-C_16_ between 0.8 and 2.0 K min^−1^ showed similar counterclockwise thermal hysteresis loops in Δ*ε*(314 nm)/temperature profiles (Fig. S10a†). The *ε*(314 nm)/temperature profiles showed a small effect of cooling and heating rates (Fig. S10b†). The results indicate the interconversion between 2**A** and **B** with minimal formation of **C**, because no upward inflation associated with the formation of **C** was observed. This behavior is consistent with a small change in Δ*ε* at 5 °C, in which the negative Δ*ε* value of −300 cm^−1^ M^−1^ was not affected by the cooling rates (Fig. 6c). This phenomenon is in contrast with that observed in (*P*)-**1**-C_16_/(*M*)-**2**, which showed a monotonic decrease of Δ*ε* at 5 °C with decreasing cooling rates (Fig. 3c). Different phenomena appeared at the lower rates of 0.1 and 0.5 K min^−1^; notably, figure-eight thermal hysteresis was observed at a very low cooling and heating rate of 0.1 K min^−1^ with a crossing point of 45 °C at a Δ*ε* value −60 cm^−1^ M^−1^ (Fig. 6a).

Constant-rate cooling and heating experiments were conducted using 0.3 and 0.7 mM (*P*)-**1**/(*M*)-**2**-C_16_ (Fig. S11†). Figure-eight thermal hysteresis appeared for the 0.3 mM solution at a very low rate of 0.1 K min^−1^ with a crossing point at 40 °C with a Δ*ε* of −20 cm^−1^ M^−1^.

**Fig. 7:**
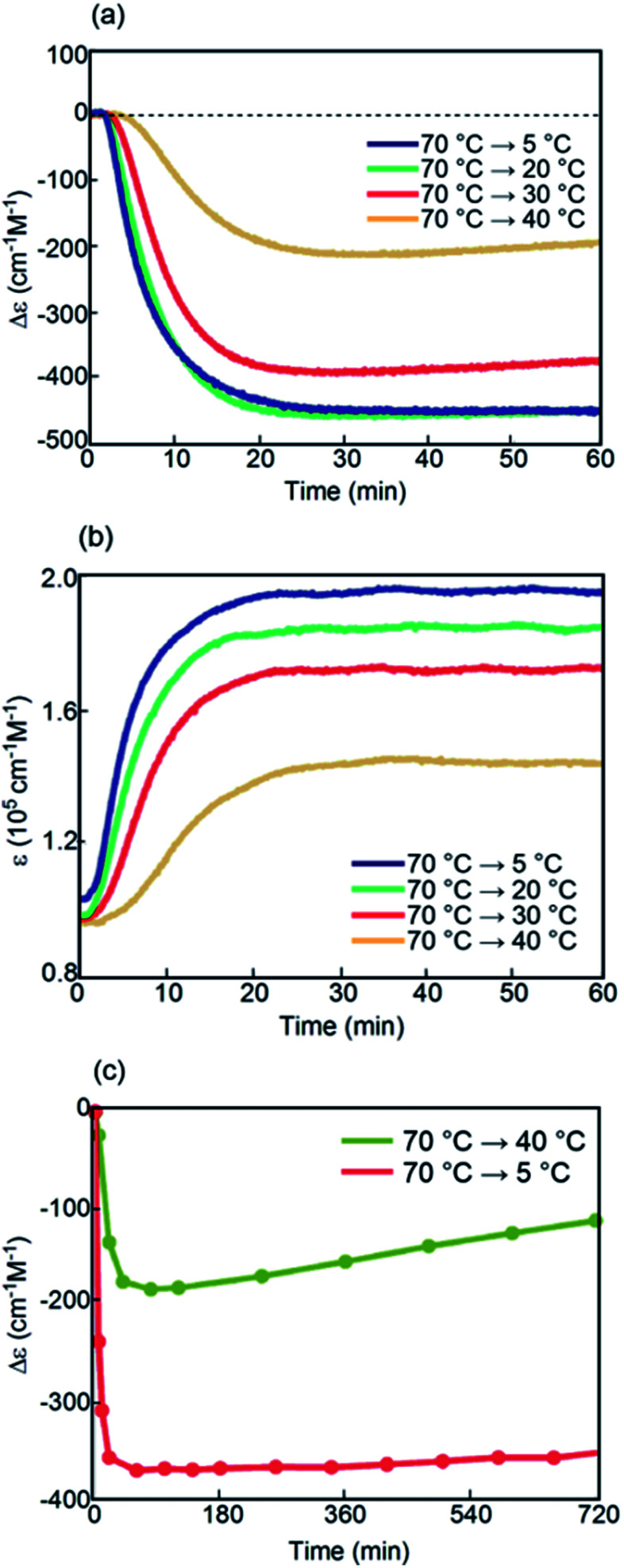
Isothermal experiment results of (*P*)-**1**/(*M*)-**2**-C_16_ in fluorobenzene (0.5 mM). (a) Δ*ε*(314 nm)/time profiles and (b) *ε*(314 nm)/time profiles at 5, 20, 30, and 40 °C. (c) Δ*ε*(314 nm)/time profiles up to 720 min at 5 and 40 °C are also shown.

Kinetic analysis of (*P*)-**1**/(*M*)-**2**-C_16_ under isothermal conditions also showed phenomena different from those observed in (*P*)-**1**-C_16_/(*M*)-**2**. A solution was cooled from 70 °C to 5 °C, and its Δ*ε* at 314 nm was monitored for 60 min; sigmoidal kinetics were obtained, which showed an initial lag time followed by a rapid decrease in Δ*ε* to −440 cm^−1^ M^−1^ in 20 min (Fig. 7a and S12a†). At 40 °C, Δ*ε* reached −200 cm^−1^ M^−1^ at 30 min and then slowly increased to −110 cm^−1^ M^−1^ after 720 min (Fig. 7a and c), which showed the initial rapid formation of **B** followed by its slow conversion to **C**. **C** is thermodynamically more stable than **B** in (*P*)-**1**/(*M*)-**2**-C_16_, which is consistent with the increase in Δ*ε* at 5 °C with the decrease in the cooling rate below 1.0 K min^−1^ (Fig. 6c).

Combinations of oligomers with terminal C_16_ alkyl groups at different positions, namely (*P*)-**1**-C_16_/(*M*)-**2** and (*P*)-**1**/(*M*)-**2**-C_16_, were compared. The figure-eight thermal hysteresis of (*P*)-**1**-C_16_/(*M*)-**2** appeared at cooling and heating rates between 1.5 and 2.5 K min^−1^, and that of (*P*)-**1**/(*M*)-**2**-C_16_ appeared at a very low rate of 0.1 K min^−1^; Δ*ε* at 5 °C showed a monotonic decrease between 0.5 and 4.0 K min^−1^ with a decreasing rate for (*P*)-**1**-C_16_/(*M*)-**2** and did not change between 0.8 and 2.0 K min^−1^ for (*P*)-**1**/(*M*)-**2**-C_16_; **B** was thermodynamically more stable than **C** in (*P*)-**1**-C_16_/(*M*)-**2**, and **C** was more stable than **B** in (*P*)-**1**/(*M*)-**2**-C_16_. The terminal C_16_ alkyl groups appeared to have significant effects on the thermodynamic and kinetic properties of aminomethylenehelicene oligomers.

### Thermal hysteresis of (*P*)-**1**-C_16_/(*M*)-**2**-C_16_

**Fig. 8:**
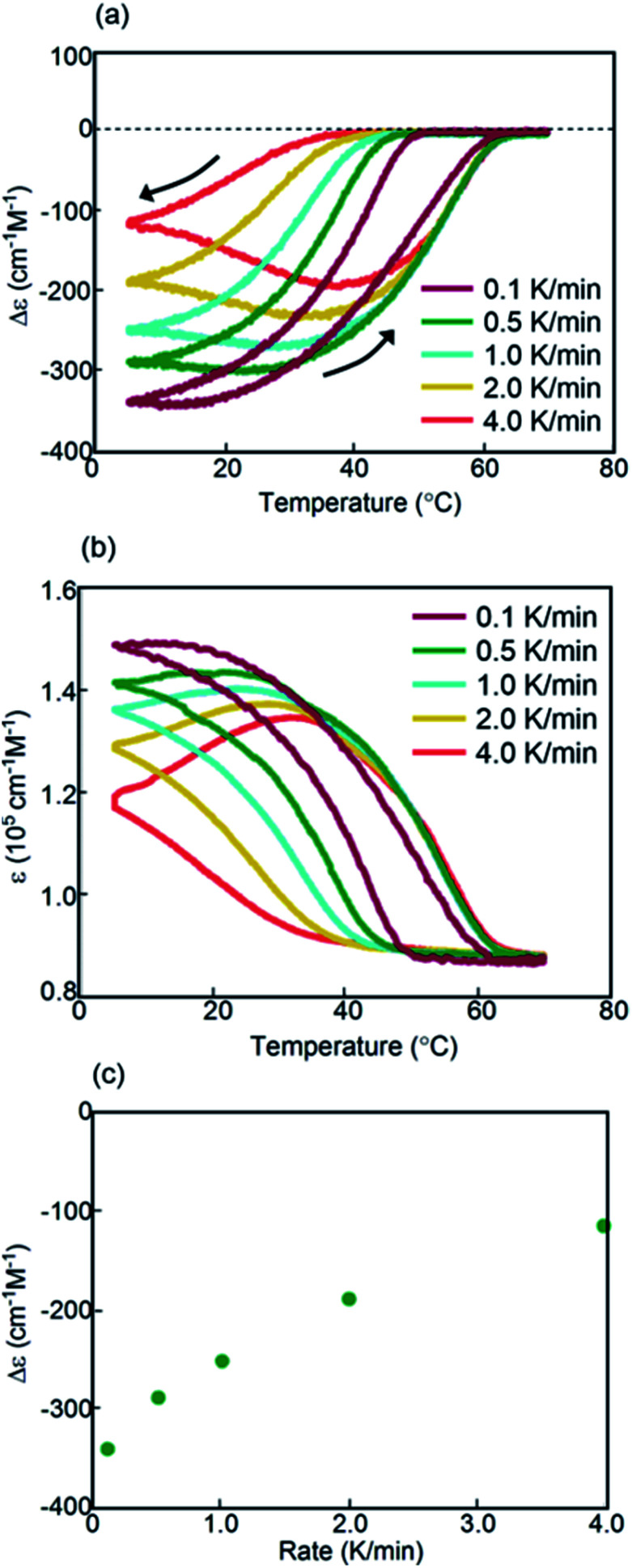
(a) Δ*ε*(314 nm)/temperature profiles and (b) *ε*(314 nm)/temperature profiles of (*P*)-**1**-C_16_/(*M*)-**2**-C_16_ in fluorobenzene (0.5 mM) at cooling and heating rates between 0.1 and 4.0 K min^−1^. The temperature was decreased from 70 °C to 5 °C and then increased from 5 °C to 70 °C. (c) Profiles of Δ*ε*(314 nm) at 5 °C as a function of cooling rate.

Another combination of (*P*)-**1**-C_16_/(*M*)-**2**-C_16_ in fluorobenzene (0.5 mM), in which both oligomers possessed terminal C_16_ alkyl groups, was examined (Fig. S13†). Constant-rate cooling and heating experiments showed thermal hysteresis loops at rates between 0.1 and 4.0 K min^−1^ with a relatively simple feature, and did not show figure-eight thermal hysteresis (Fig. 8a and b). As the cooling rate decreased, Δ*ε* at 5 °C decreased, and a Δ*ε* value of −340 cm^−1^ M^−1^ was obtained at a rate of 0.1 K min^−1^ (Fig. 8c). This is due to the higher thermodynamic stability of **B** than of **C**. The thermal hysteresis in *ε*(314 nm)/temperature profiles showed the increase of *ε* at 5 °C with the decreasing cooling rate (Fig. 8b). These results are consistent with the involvement of interconversion between 2**A** and **B** with minimal formation of **C** because no upward inflation associated with the formation of **C** was observed.

Kinetic analysis of (*P*)-**1**-C_16_/(*M*)-**2**-C_16_ under isothermal conditions provided sigmoidal curves (Fig. S14†). At 20 and 30 °C, similar Δ*ε*/time profiles were obtained.

At 5 °C, an initial decrease of Δ*ε* occurred at a similar rate to those in 20 and 30 °C experiments; the decrease was retarded upon reaching −230 cm^−1^ M^−1^ at 60 min.

### Thermal hysteresis of (*P*)-**1**/(*M*)-**2**

The thermal hysteresis of (*P*)-**1**/(*M*)-**2** without the C_16_ groups was examined previously^37–39^ and was examined here again to compare with that of (*P*)-**1**-C_16_/(*M*)-**2** and related systems possessing terminal C_16_ alkyl groups.

**Fig. 9:**
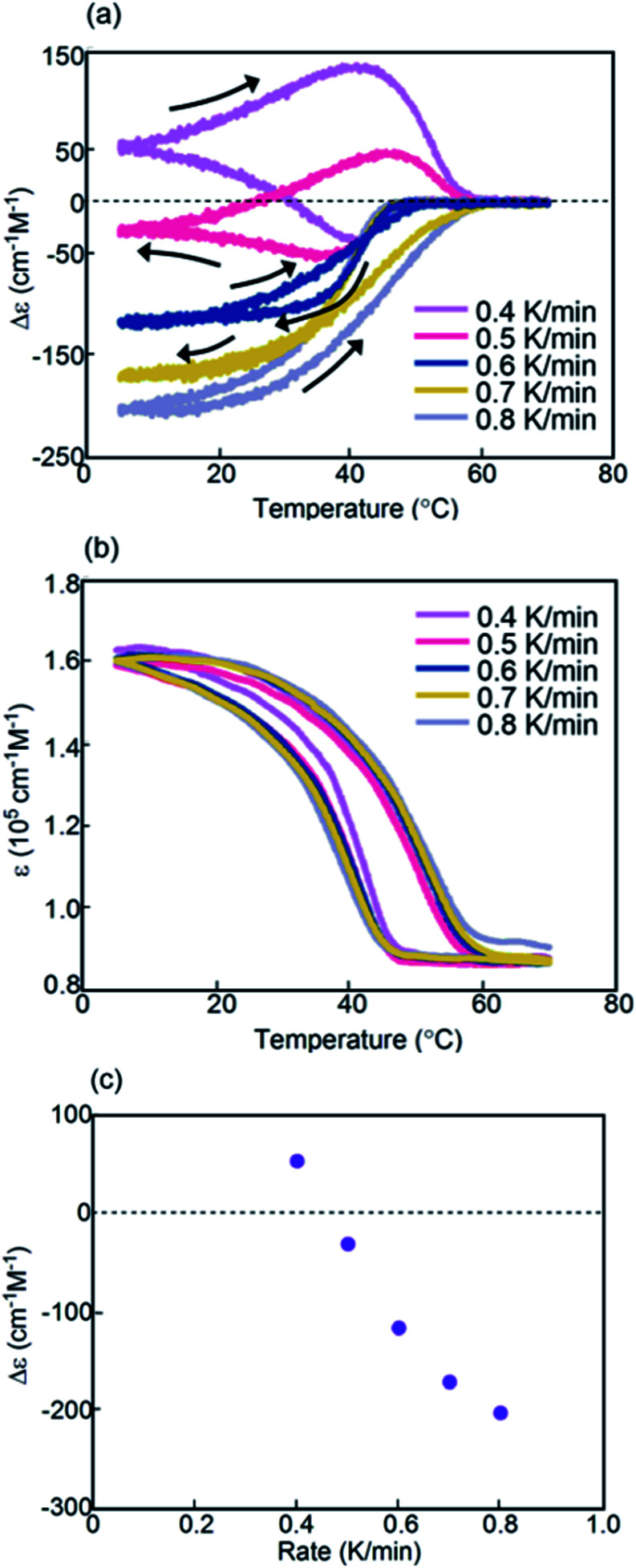
(a) Δ*ε*(314 nm)/temperature profiles and (b) *ε*(314 nm)/temperature profiles of (*P*)-**1**/(*M*)-**2** in fluorobenzene (0.5 mM) at cooling and heating rates of 0.4 to 0.8 K min^−1^. The temperature was decreased from 70 °C to 5 °C and then increased from 5 °C to 70 °C. (c) Profiles of Δ*ε*(314 nm) at 5 °C as a function of cooling rate.

At cooling and heating rates of 0.4 and 0.5 K min^−1^, clockwise thermal hysteresis appeared in the domain close to 0 cm^−1^ M^−1^ (Fig. 9a). At higher rates between 0.7 and 0.8 K min^−1^, counterclockwise thermal hysteresis appeared in the negative Δ*ε* domains. Figure-eight thermal hysteresis appeared at 0.6 K min^−1^ with a crossing point at 42 °C and a Δ*ε* value of −40 cm^−1^ M^−1^. These results confirmed that figure-eight thermal hysteresis appeared in the intermediate state of clockwise and counterclockwise thermal hystereses and at the domains close to a Δ*ε* value of 0 cm^−1^ M^−1^, as noted in (*P*)-**1**-C_16_/(*M*)-**2** (Fig. 3a). The figure-eight thermal hysteresis loop of (*P*)-**1**/(*M*)-**2** was a distorted sigmoid, which was similar to sigmoids appearing in the 0.7 and 0.8 K min^−1^ experiments. An upward inflation at 5 °C did not appear, in contrast to the behavior in (*P*)-**1**-C_16_/(*M*)-**2** (Fig. 3a).

A different phenomenon observed for (*P*)-**1**/(*M*)-**2** from (*P*)-**1**-C_16_/(*M*)-**2** is that the decrease of the cooling and heating rate converted counterclockwise thermal hysteresis to clockwise (Fig. 9a), which is in contrast to that for (*P*)-**1**-C_16_/(*M*)-**2** (Fig. 3a). Another difference is that Δ*ε* at 5 °C showed a monotonous increase with a decrease of the cooling rate for (*P*)-**1**/(*M*)-**2** (Fig. 9c), which is in contrast to that of (*P*)-**1**-C_16_/(*M*)-**2** (Fig. 3c). The results are explained by the higher thermodynamic stability of **C** than of **B** in (*P*)-**1**/(*M*)-**2**, which is consistent with that in our previous study.^37–39^

**Fig. 10:**
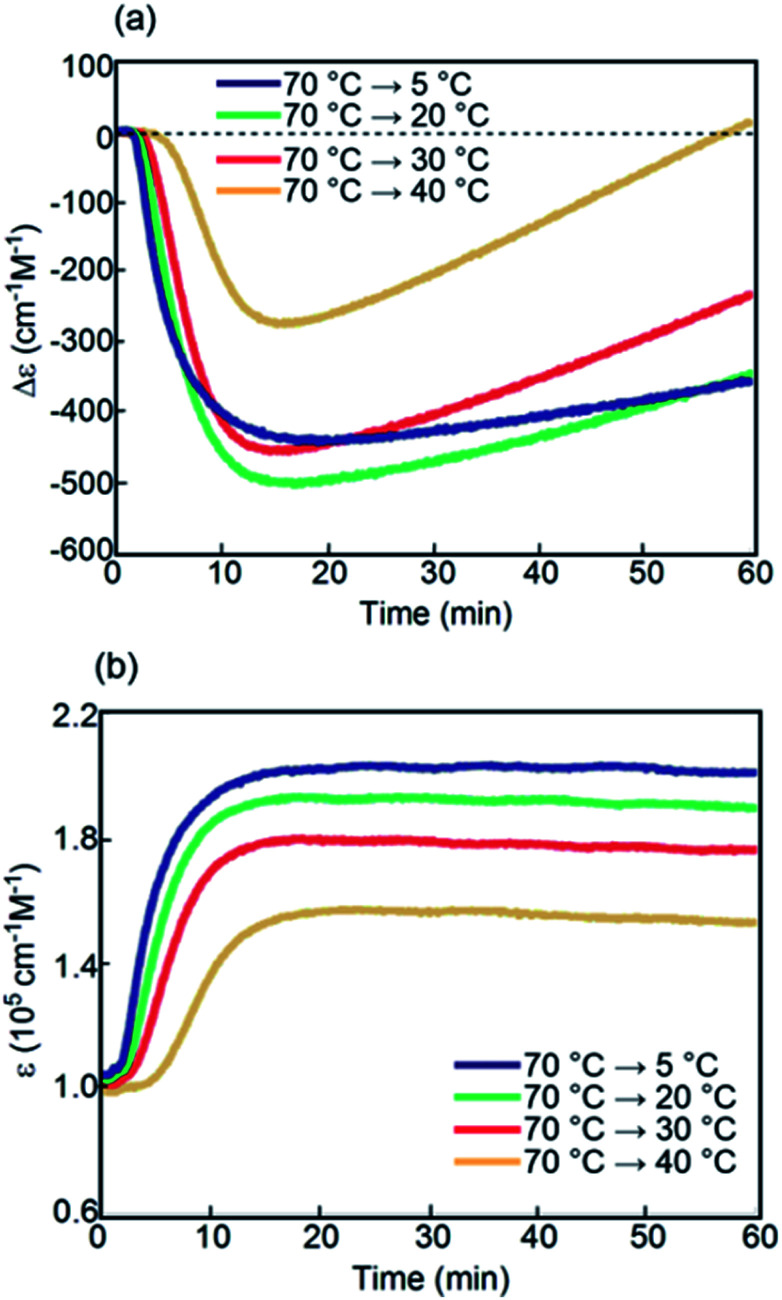
(a) Δ*ε*(314 nm)/time profiles and (b) *ε*(314 nm)/time profiles for (*P*)-**1**/(*M*)-**2** in fluorobenzene (0.5 mM) at 5, 20, 30, and 40 °C.

Kinetic analysis of (*P*)-**1**/(*M*)-**2** under isothermal conditions between 5 and 40 °C provided sigmoidal curves in Δ*ε*(314 nm)/temperature profiles. Δ*ε* rapidly decreased initially and then increased; this indicated the initial formation of **B** followed by the conversion to **C**, which is consistent with previous observations (Fig. 10).^37–39^ At 5, 20, and 30 °C, similar curves were obtained. At 40 °C, a slower decrease in Δ*ε* followed by a significant increase was observed, which resulted in a positive Δ*ε* of +10 cm^−1^ M^−1^ at 60 min.

Figure-eight thermal hysteresis appearing at constant-rate cooling and heating rates depends on the structure of helicene oligomers: (*P*)-**1**-C_16_/(*M*)-**2** at 1.5 and 2.5 K min^−1^, (*P*)-**1**/(*M*)-**2**-C_16_ at 0.1 K min^−1^, and (*P*)-**1**/(*M*)-**2** at 0.6 K min^−1^. It was noted that (*P*)-**1**-C_16_/(*M*)-**2** showed figure-eight thermal hysteresis in a broader range of rates compared to (*P*)-**1**/(*M*)-**2**. The terminal C_16_ alkyl groups significantly affected the dynamics of helicene oligomers.

### Discussion

Figure-eight hysteresis appears under various conditions in nature as described in the introduction, and is considered to be a complex phenomenon. This work shows that figure-eight thermal hysteresis is exhibited by a noncovalent chemical reaction with molecules dispersed in solution, and Δ*ε* (output) in response to a temperature change (input) can be related to the concentrations of the molecules. Figure-eight thermal hysteresis clearly appeared in (*P*)-**1**-C_16_/(*M*)-**2** and in a distorted form in (*P*)-**1**/(*M*)-**2** with the following features in experiments: (1) it appears in the intermediate state between counterclockwise and clockwise thermal hystereses; (2) it occurs in the domain close to Δ*ε* of 0 cm^−1^ M^−1^; (3) it involves significant changes in Δ*ε* at 5 °C depending on cooling and heating rate; (4) it depends on thermal history.

**Fig. 11:**
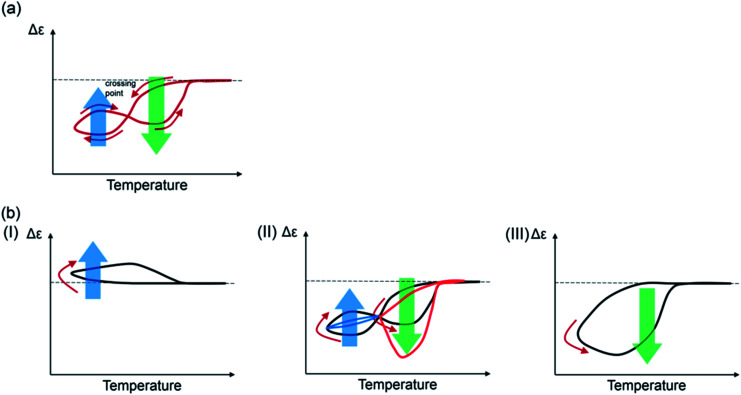
(a) Model of figure-eight thermal hysteresis (red lines and arrows) of (*P*)-**1**-C_16_/(*M*)-**2** during structural changes between 2**A**, **B**, and **C**. Bold arrows indicate self-catalytic reactions to form **B** (green) and **C** (blue). (b) Rate dependence of thermal hysteresis. At high cooling and heating rates, clockwise thermal hysteresis appears (I); at low rates, counterclockwise thermal hysteresis appears (III). Figure-eight thermal hysteresis is an intermediate phenomenon (II).

A qualitative model for the figure-eight thermal hysteresis of (*P*)-**1**-C_16_/(*M*)-**2**, which involves the competitive self-catalytic reactions to form **B** and **C**, is provided by Δ*ε*/temperature profiles (Fig. 11a). It is critical that Δ*ε* increases at lower ends of the temperature changes exhibiting upward inflation, which is derived from the formation of **C** (bold blue arrows). At higher temperatures, the formation of **B** predominates with minimum formation of **C**, which shows downward inflation (bold green arrows). Figure-eight thermal hysteresis is derived from the different temperature dependencies of the self-catalytic reactions to form **B** and **C**. Quantitative analysis for the figure-eight thermal hysteresis is a subject for research in the future.^40^

The figure-eight thermal hysteresis of (*P*)-**1**-C_16_/(*M*)-**2** appeared in an intermediate state between counterclockwise thermal hysteresis at low cooling and heating rates and clockwise thermal hysteresis at high rates (Fig. 11b). Rate dependency may be a common feature of various figure-eight hysteresis phenomena observed in different fields.^35,36^

Another interesting phenomenon in this study is the effect of terminal C_16_ alkyl groups exhibiting significantly different properties of (*P*)-**1**-C_16_/(*M*)-**2**, (*P*)-**1**/(*M*)-**2**-C_16_, (*P*)-**1**-C_16_/(*M*)-**2**-C_16,_ and (*P*)-**1**/(*M*)-**2**. Figure-eight thermal hysteresis appeared when using (*P*)-**1**-C_16_/(*M*)-**2**, (*P*)-**1**/(*M*)-**2**-C_16,_ and (*P*)-**1**/(*M*)-**2** and not when using (*P*)-**1**-C_16_/(*M*)-**2**-C_16_. Suitable cooling and heating rates in 0.5 mM fluorobenzene solutions are between 1.5 and 2.5 K min^−1^ for (*P*)-**1**-C_16_/(*M*)-**2**, at a very low rate of 0.1 K min^−1^ for (*P*)-**1**/(*M*)-**2**-C_16_, and 0.6 K min^−1^ with a distorted shape for (*P*)-**1**/(*M*)-**2**. Note that (*P*)-**1**-C_16_/(*M*)-**2** exhibited figure-eight thermal hysteresis over a broader range.

The relative thermodynamic stability of **B** and **C** significantly changed depending on C_16_ alkyl groups, as indicated by kinetic analysis and the effect of the cooling rate on Δ*ε* at 5 °C. A higher thermodynamic stability of **B** than of **C** was observed when using (*P*)-**1**-C_16_/(*M*)-**2** and (*P*)-**1**-C_16_/(*M*)-**2**-C_16_. A higher thermodynamic stability of **C** than of **B** was observed when using (*P*)-**1**/(*M*)-**2**-C_16_ and (*P*)-**1**/(*M*)-**2**. Thus, the terminal C_16_ alkyl groups significantly affect the thermodynamic and kinetic properties of enantiomer mixtures of aminomethylenehelicene oligomers.

The effect of long alkyl groups on the chemical reaction of molecules dispersed in solution, as shown in this study, is interesting. The interactions between long chain alkyl groups at the molecular level are considered weak, and generally have small effects on chemical reactions. This behavior is in contrast with the significant effect of long alkyl groups on self-assembly materials for liquid crystals and membranes in the bulk, in which multiple interactions between long alkyl groups occur. The notable effect of the terminal C_16_ alkyl groups observed in this study at the molecular level can be ascribed to the involvement of different conformations or structures of the C_16_ moiety, for example to form helical structures.^41^ It is reasonable to consider that the conformation can be affected by the structure of the hetero-double-helix and also by the location of the C_16_ moiety.

## Conclusions

To summarize, a mixture of aminomethylenehelicene (*P*)-tetramer (*P*)-**1**-C_16_ and (*M*)-pentamer (*M*)-**2**-C_16_ with terminal C_16_ alkyl groups exhibited figure-eight thermal hysteresis, which was clearly observed when using (*P*)-**1**-C_16_/(*M*)-**2**. The figure-eight thermal hysteresis was analyzed employing profiles of Δ*ε* and concentration (output) as a function of temperature (input). Such systems may have applications in optical devices based on the Δ*ε* output and in material devices based on the concentration output, which sensitively respond to the thermal environment and history. Figure-eight hysteresis was expressed by concentrations of substances and products, and was explained by a concise mechanistic model. This work can be a basis to understand and compare the hysteresis phenomena in different fields.

## Conflicts of interest

There are no conflicts to declare.

## Supplementary Material

SC-011-C9SC06496F-s001
