# Supplementary material for: Figure-eight thermal hysteresis of aminomethylenehelicene oligomers with terminal C16 alkyl groups during hetero-double-helix formation
Source: Chem Sci. 2020 Feb 26;11(12):3290–300. doi: 10.1039/c9sc06496f (PMC8157274; doi:10.1039/c9sc06496f)
Supplement: SC-011-C9SC06496F-s001 [file SC-011-C9SC06496F-s001.pdf]

Electronic Supplementary Material (ESI) for Chemical Science.
This journal is © The Royal Society of Chemistry 2020

Figure-eight Thermal Hysteresis of Aminomethylenehelicene Oligomers with Terminal C_{16} Alkyl Groups during Hetero-double-helix Formation

Tsukasa Sawato, Rina Iwamoto and Masahiko Yamaguchi*

Department of Organic Chemistry, Graduate School of Pharmaceutical Sciences,

Tohoku University, Aoba, Sendai 980-8578, Japan

*E-mail: yama@m.tohoku.ac.jp, Fax: (+81) 22-795-6811

Supporting Information

Table of Content

1. Synthesis
2. (*P*)-**1**-C_{16}/(*M*)-**2** system
3. (*P*)-**1**/(*M*)-**2**-C_{16} system
4. (*P*)-**1**-C_{16}/(*M*)-**2**-C_{16} system
5. Comparison of (*P*)-**1**/(*M*)-**2** system
6. References

1. Synthesis

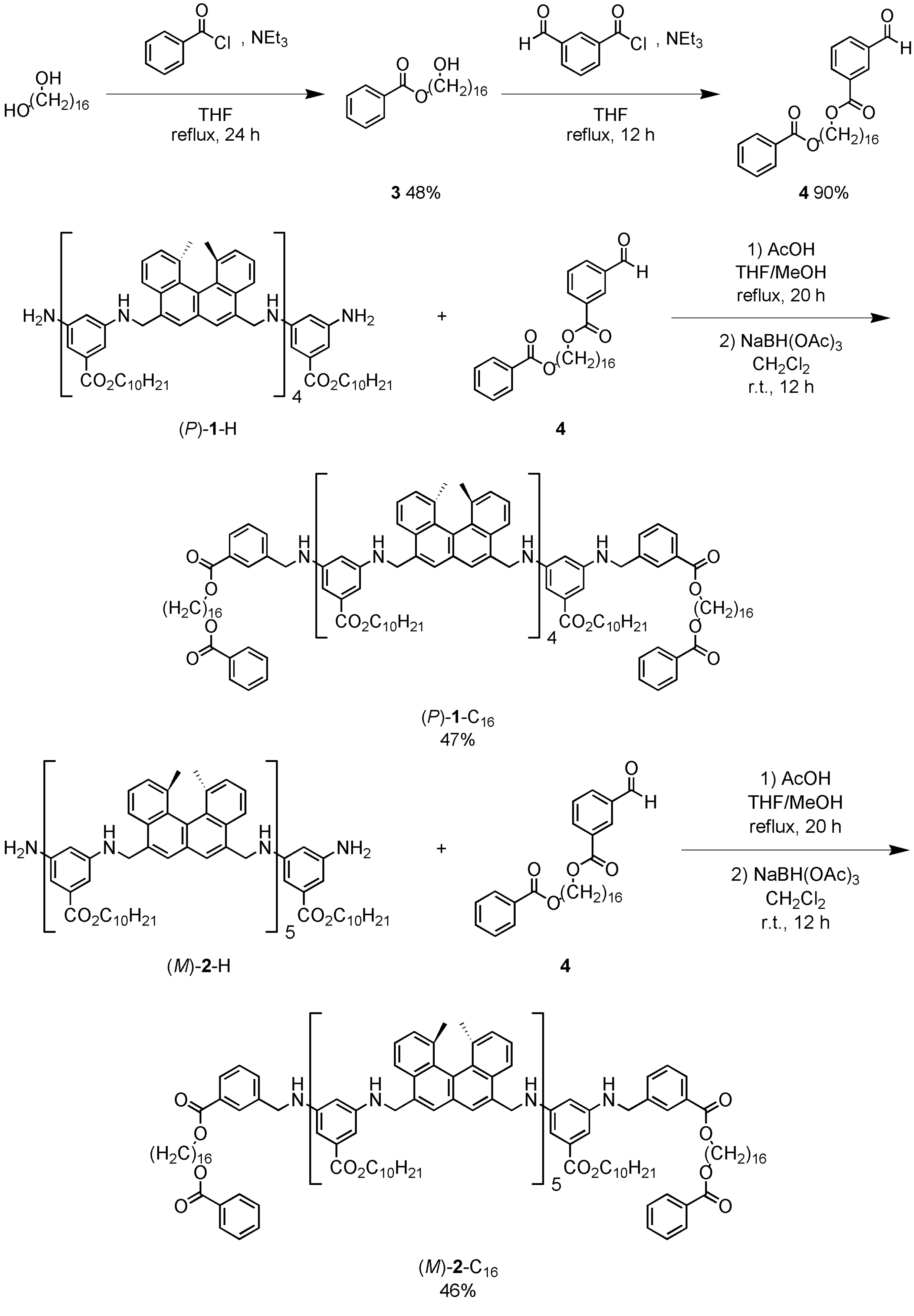

Scheme S1. Synthesis of (*P*)-**1**-C_{16} and (*M*)-**2**-C_{16}.

1,16-Hexadecandiol Benzoyl Ester, 3. Under an argon atmosphere, a mixture of 1,16-decandiol (207 mg, purity 97%, 0.774 mmol), benzoyl chloride (90 μL, 0.775 mmol), triethylamine (0.1 mL) and tetrahydrofuran (10 mL) was heated under reflux for 24 h. The mixture was cooled to room temperature, and saturated sodium hydrogen carbonate was added. The organic materials were extracted with ethyl acetate. The organic layer was washed with water and brine, and dried over sodium sulfate. The solvent was evaporated under reduced pressure, and separation by silica gel chromatography gave **3** as white crystal (135 mg, 0.372 mmol, 48%). LRMS (EI, 70 eV) *m/z*: 362 ($[M]^+$). HRMS *m/z* Calcd for $C_{23}H_{38}O_3^+$: 362.2821. Found: 362.2861. Anal. ($C_{23}H_{38}O_3$) Calcd for: C, 76.20; H, 10.56%. Found: C, 76.04; H, 10.41%. Mp 61 °C. IR (KBr) 3420, 2921, 2848, 1717, 1464 1119, 712 cm^{-1}. ^{1}H NMR (400 MHz, $CDCl_3$) δ 1.26-1.48 (24H, m), δ 1.53-1.60 (2H, quin, J = 7.6 Hz), δ 1.73-1.80 (2H, quin, J = 7.6 Hz), δ 3.62-3.66 (2H, t, J = 6.8 Hz), δ 4.30-4.33 (2H, t, J = 6.8 Hz), δ 7.42-7.46 (2H, t, J = 8.0 Hz) , δ 7.54-7.57 (1H, t, J = 7.2 Hz), δ 8.03-8.06 (2H, d, J = 7.2 Hz). ^{13}C NMR (100 MHz, $CDCl_3$) 25.7, 26.0, 28.7, 29.3, 29.4, 29.5, 29.56, 29.58, 29.59, 29.61, 29.63, 32.8, 63.1, 65.1, 128.3, 129.5, 130.5, 132.8, 166.7.

1,16-Hexadecandiol 3-formylbenzoyl Benzoyl Ester, 4. Under an argon atmosphere, a mixture of **3** (195 mg, 0.538 mmol), 3-formylbenzoyl chloride (187 mg, 1.11 mmol), triethylamine (0.3 mL) and tetrahydrofuran (7 mL) was heated under reflux for 12 h. The mixture was cooled to room temperature, and saturated sodium hydrogen carbonate was added. The organic materials were extracted with ethyl acetate. The organic layer was washed with water and brine, and dried over sodium sulfate. The solvent was evaporated under reduced pressure, and separation by silica gel chromatography gave **4** as white crystal (239 mg, 0.483 mmol, 90%). LRMS (EI,70 eV) *m/z*: 494 ($[M]^+$). HRMS *m/z* Calcd for $^+$: 494.3032. Found: 494.3032. Anal. ($C_{31}H_{42}O_5$) Calcd for: C, 75.27; H, 8.56%. Found: C, 75.29; H, 8.59%. Mp 50 °C. IR (KBr) 2916, 2850, 1720, 1605, 1286 cm^{-1}. ^{1}H NMR (400 MHz, $CDCl_3$) δ 1.26-1.46 (24H, m), δ 1.73-1.82 (4H, m), δ 4.30-4.38 (4H, m), δ 7.42-7.46 (2H, t, J = 7.2 Hz), δ 7.53-7.57 (1H, t, J = 7.2 Hz), δ 7.61-7.65 (1H, t, J = 7.6 Hz) , δ 8.03-8.06 (2H, d7, J = 7.2 Hz), δ 8.07-8.10 (1H, d, J = 8.0 Hz), δ 8.29-8.32 (1H, d, J = 8.0 Hz) , δ 8.52 (1H, s) , δ 10.9 (1H, s). ^{13}C NMR (100 MHz, $CDCl_3$) 25.99, 26.02, 28.65, 28.70, 29.3, 29.5, 29.58, 29.62, 29.7, 128.3, 129.2, 129.5, 130.5, 131.2, 132.8, 133.0, 135.2, 136.5, 165.5, 166.7, 191.5.

(*P*)-Aminomethylenehelicene tetramer with C_{16} alkyl groups, (*P*)-1-C_{16}.

Under an argon atmosphere, a mixture of ; (*P*)-**1**-H[S1] (93.8 mg, 36.3 μmol), **4** (53.0 mg, 0.107 mmol), acetic acid (42 μL, 0.734 mmol), tetrahydrofuran (2.8 mL) and methanol (1.4 mL) was heated under reflux for 24 h. After cooling to room temperature, the solvent was evaporated under reduced pressure. Under an argon atmosphere, to the mixture in dichloromethane was added sodium triacetoxyborohydride (226 mg, 1.07 mmol) at 0 °C, and the mixture was stirred vigorously at room

temperature for 12 h. The reaction was quenched by adding saturated aqueous sodium hydrogen carbonate, and the organic materials were extracted with dichloromethane. The organic layer was washed with water and brine, and dried over sodium sulfate. The solvent was evaporated under reduced pressure, and separation by silica gel chromatography and GPC gave (*P*)-**1**-C_{16} as orange crystals (60.4 mg, 17.1 μmol, 47%). MS (MALDI TOF) *m/z* Calcd for $C_{235}H_{288}N_{10}NaO_8$ ($[M+Na]^+$): 3561.18. Found: 3561.67. Anal. ($C_{235}H_{288}N_{10}O_8$) Calcd for: C, 79.71; H, 8.20; N, 3.96%. Found: C, 79.63; H, 8.14; N, 3.93%. Mp 109-115 °C. $[\alpha]^{30}{}_D$ −80 (*c* 0.10, $CHCl_3$). IR (KBr) 3421, 2924, 2852, 1716, 1606 cm^{-1}. ^1H NMR (400 MHz, $CDCl_3$) δ 0.81-0.86 (15H, m), δ 1.21-1.44 (118H, m), δ 1.59-1.78 (26H, m), δ 1.90-1.91 (24H, m), δ 4.10 (10H, m), δ 4.17-4.31 (18H, m), δ 4.72-4.83 (16H, m), δ 6.02-6.20 (5H, m), δ 6.74 (2H, s), δ 6.83-6.87 (8H, m) , δ 7.35-7.55 (26H, m), δ 7.68 (8H, s), δ 7.85-7.87 (2H, d, 8.0 Hz), δ 7.96-8.04 (14H, m). ^{13}C NMR (100 MHz, $CDCl_3$) 14.1, 22.6, 23.5, 25.96, 26.01, 28.65, 28.67, 29.2, 29.3, 29.5, 29.57, 29.59, 29.63, 29.7, 31.8, 46.5, 47.8, 64.9, 65.1, 65.2, 100.8, 103.8, 104.2, 120.8, 125.3, 126.0, 128.28, 128.37, 128.5, 128.6, 129.5, 130.5, 130.6, 130.8, 131.5, 131.7, 131.8, 132.25, 132.34, 132.8, 133.16, 133.22, 136.8, 139.6, 148.9, 149.2, 149.3, 166.5, 166.7, 167.2, 167.3.

(*M*)-Aminomethylenehelicene pentamer with C_{16} alkyl groups, (*M*)-2-C_{16}.

Under an argon atmosphere, a mixture of (*M*)-**2**-H[S1] (281 mg, 89.0 μmol), **4** (239 mg, 0.483 mmol), acetic acid (150 μL, 2.62 mmol), tetrahydrofuran (14 mL) and methanol (7 mL) was heated under reflux for 24 h. After cooling to room temperature, the solvent was evaporated under reduced pressure. Under an argon atmosphere, to the mixture in dichloromethane was added sodium triacetoxyborohydride (566 mg, 2.67 mmol) at 0 °C, and the mixture was stirred vigorously at room temperature for 12 h. The reaction was quenched by adding saturated aqueous sodium hydrogen carbonate, and the organic materials were extracted with dichloromethane. The organic layer was washed with water and brine, and dried over sodium sulfate. The solvent was evaporated under reduced pressure, separation by silica gel chromatography and GPC gave (*M*)-**2**-C_{16} as orange crystals (168 mg, 40.8 μmol, 46%). MS (MALDI TOF) *m/z* Calcd for $C_{274}H_{332}N_{12}O_{20}$ ($[M+Na]^+$) : 4133.52. Found: 4133.56. Anal. ($C_{274}H_{332}N_{12}O_{20}$) Calcd for: C, 80.00; H, 8.13; N, 4.09%. Found: C, 79.78; H, 8.29; N, 4.06%. Mp 148-151 °C. $[\alpha]^{30}{}_D$ +46 (*c* 0.10, $CHCl_3$). IR (KBr) 3420, 2924, 2853, 1716, 1606 cm^{-1}. ^1H NMR (400 MHz, $CDCl_3$) δ 0.81-0.86 (18H, m), δ 1.21-1.46 (132H, m), δ 1.58-1.78 (24H, m), δ 1.90-1.91 (30H, m), δ 4.08 (12, s), δ 4.17-4.31 (20H, m), δ 4.67-4.74 (20H, m), δ 6.00-6.12 (6H, m), δ 6.72 (2H, s), δ 6.82-6.84 (10H, m) , δ 7.33-7.55 (30H, m), δ 7.64 (10H, s), δ 7.84-7.86 (2H, d, 7.6 Hz), δ 7.97-8.04 (16H, m). ^{13}C NMR (100 MHz, $CDCl_3$) 14.1, 22.6, 23.5, 25.9, 26.0, 28.6, 29.2, 29.3, 29.49, 29.52, 29.58, 29.63, 29.66, 29.71, 31.8, 31.9, 46.39, 47.8, 64.9, 65.1, 65.2, 100.8, 100.9, 103.8, 104.2, 104.7, 120.8, 125.2, 126.0, 128.3, 128.4, 128.5, 128.6, 129.5, 130.45, 130.54, 130.8,

131.4, 131.7, 131.8, 132.26, 132.32, 132.8, 133.2, 136.8, 139.6, 148.9, 149.16, 149.24, 166.5, 166.7, 167.2, 167.3.

2. (*P*)-1-C_{16}/(*M*)-2 system

<Formation of hetero-double-helix B>

A mixture of (*P*)-**1**-C_{16} and (*M*)-**2** in fluorobenzene (0.5 mM) was heated at 70 °C for 10 min, and cooled to 40 °C for 140 min, cooled to 20 °C for 1020 min, and cooled to 5 °C for 60 min. CD and UV-vis analyses were conducted at the temperatures (Figure 2). The same experiments were conducted in fluorobenzene at 1.0 and 1.2 mM and also in 1,3-difluorobenzene at 0.5 mM (Figures S1a, S1b, and S1c). DLS analysis (fluorobenzene, 0.5 mM) was also conducted at 20 and 70 °C (Figure S1d and S1e).

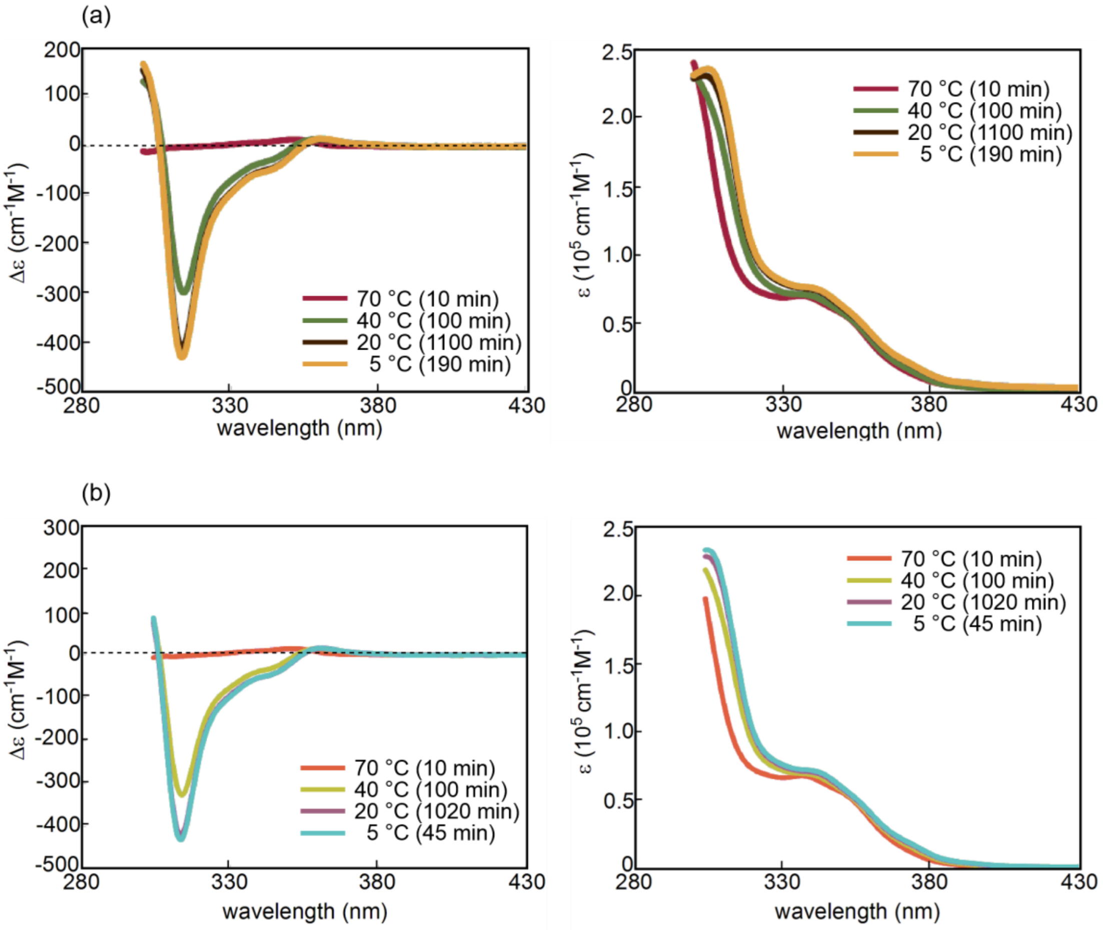

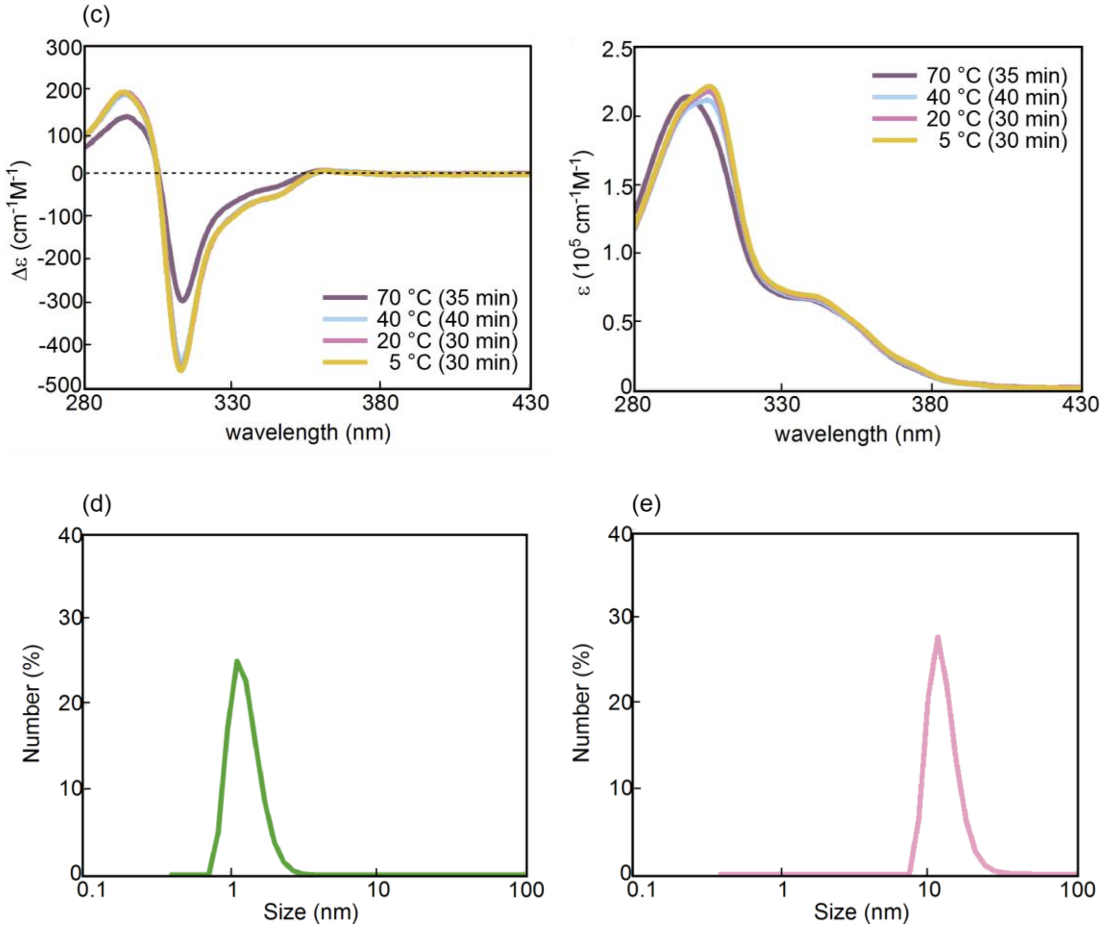

Figure S1. CD and UV-vis spectra of (*P*)-**1**-C_{16}/(*M*)-**2** in fluorobenzene at 70, 40, 20, and 5 °C at the concentrations of (a) 1.0 mM and (b) 1.2 mM. (c) CD and UV-vis spectra of (*P*)-**1**-C_{16}/(*M*)-**2** in 1,3-difluorobenzene (0.5 mM) at 70, 40, 20, and 5 °C. DLS analysis of (*P*)-**1**-C_{16}/(*M*)-**2** in fluorobenzene (0.5 mM) at (d) 70 °C and (e) 20 °C.

The above experiments determined the spectra at the S-state of hetero-double-helix **B** with $\Delta\varepsilon_B$ −430 $cm^{-1}M^{-1}$ and ε_B 1.6×10^5 $cm^{-1}M^{-1}$, in which all the molecules are **B**.

<Relation of Δε/ε and concentration>

The analysis by Δε (314 nm)/temperature and ε (314 nm)/temperature profiles provides concentration/temperature profiles, which assumes the formation of 2**A**, **B**, and **C**.

Δε at 314 nm can be related to the concentrations of **A**, **B**, and **C** by

$[\mathbf{A}]_0\Delta\varepsilon = 2(\Delta\varepsilon_B[\mathbf{B}] + \Delta\varepsilon_C[\mathbf{C}]) + \Delta\varepsilon_A[\mathbf{A}]$ (1)

$[\mathbf{A}]_0 = [\mathbf{A}] + 2[\mathbf{B}] + 2[\mathbf{C}]$ (2)

in which $\Delta\varepsilon_A$, $\Delta\varepsilon_B$, and $\Delta\varepsilon_C$ are Δε of **A**, **B**, and **C** in the S-states, respectively. The weak Cotton effect of 2**A** assumed $\Delta\varepsilon_A = 0$ $cm^{-1}M^{-1}$, and the enantiomeric nature of **B** and **C** assumed $\Delta\varepsilon_B = -\Delta\varepsilon_C$. Then, the equation 1 can be expressed by

$\Delta\varepsilon = 2\Delta\varepsilon_B([\mathbf{B}] - [\mathbf{C}])/[\mathbf{A}]_0$. (3)

UV absorption ε at 314 nm is also related to the concentrations of **A**, **B**, and **C** by

$[\mathbf{A}]_0\varepsilon = \varepsilon_A[\mathbf{A}] + 2(\varepsilon_B[\mathbf{B}] + \varepsilon_C[\mathbf{C}])$ (4)

in which ε_A, ε_B, and ε_C are ε of **A**, **B**, and **C** at the S-states, respectively.

The equation 4 can be expressed by

$\varepsilon = \{\varepsilon_A[\mathbf{A}] + 2\varepsilon_B([\mathbf{B}] + [\mathbf{C}])\}/[\mathbf{A}]_0.$ (5)

From equations 2, 3, and 5, unique [**A**], [**B**], and [**C**] are determined.

$[\mathbf{A}] = (\varepsilon - \varepsilon_B)[\mathbf{A}]_0/(\varepsilon_A - \varepsilon_B)$

$[\mathbf{B}] = \{(\Delta\varepsilon/\Delta\varepsilon_B) + (\varepsilon_A - \varepsilon)/(\varepsilon_A - \varepsilon_B)\}[\mathbf{A}]_0/4$

$[\mathbf{C}] = \{(\varepsilon_A - \varepsilon)/(\varepsilon_A - \varepsilon_B) - (\Delta\varepsilon/\Delta\varepsilon_B)\}[\mathbf{A}]_0/4$

Concentration/temperature and concentration/time profiles are obtained employing the following values: $\Delta\varepsilon_B = -\Delta\varepsilon_C = -430$ cm^{-1}M^{-1}, $\Delta\varepsilon_A = 0$ cm^{-1}M^{-1}, $\varepsilon_A = 0.9\times10^5$ cm^{-1}M^{-1}, $\varepsilon_B = \varepsilon_C = 1.6\times10^5$ cm^{-1}M^{-1}, and $[\mathbf{A}]_0 = 0.5$ mM.

Concentration/temperature and concentration/time profiles are converted to content/temperature and content/time profiles, respectively, in which the contents (%) of **B** and **C** are calculated twice the concentrations [**B**] and [**C**], respectively (Figure 3c).

<Constant-rate cooling and heating experiments>

A mixture of (*P*)-**1**-C_{16} and (*M*)-**2** in fluorobenzene (0.5 mM) was heated at 70 °C for 10 min, cooled to 5 °C, and heated to 70 °C at the rates shown in Figures 3a and 3b, during which $\Delta\varepsilon$ at 314 nm and ε at 314 nm were monitored. Content/temperature profiles are also shown (Figures 3d and S2). Experiments were also conducted at the concentration of 0.4 and 0.6 mM (Figure S3)

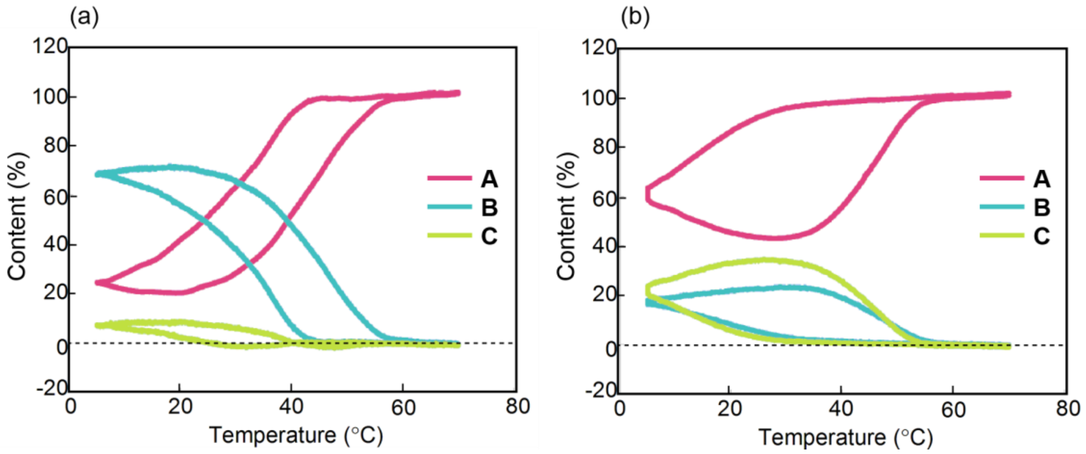

Figure S2. Constant-rate cooling and heating experiments of (*P*)-**1**-C_{16}/(*M*)-**2** in fluorobenzene (0.5 mM) shown by content/temperature profiles at the rates of (a) 0.5 K/min and (b) 4.0 K/min. Temperature was decreased from 70 °C to 5 °C and then increased from 5 °C to 70 °C.

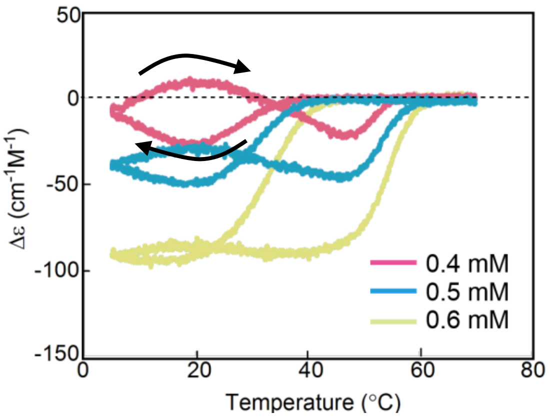

Figure S3. Effect of concentration in the constant-rate cooling and heating experiments of (*P*)-**1**-C_{16}/(*M*)-**2** in fluorobenzene (0.4, 0.5, and 0.6 mM) shown by Δε/temperature profiles at the rate of 2.0 K/min.

<Isothermal experiments>

A mixture of (*P*)-**1**-C_{16} and (*M*)-**2** in fluorobenzene (0.5 mM) was heated at 70 °C for 10 min, immediately cooled to 40, 30, 20, or 5 °C, and Δε and ε at 314 nm were monitored at the temperatures for 60 min at 5 sec intervals (Figures 4a and 4b). CD and UV-Vis spectra were also obtained up to 640 min (Figures S4a, S4b, S4c, and S4d). Long experiment was also conducted up to 1680 min (Figure S4e), from which Figure 4e was obtained.

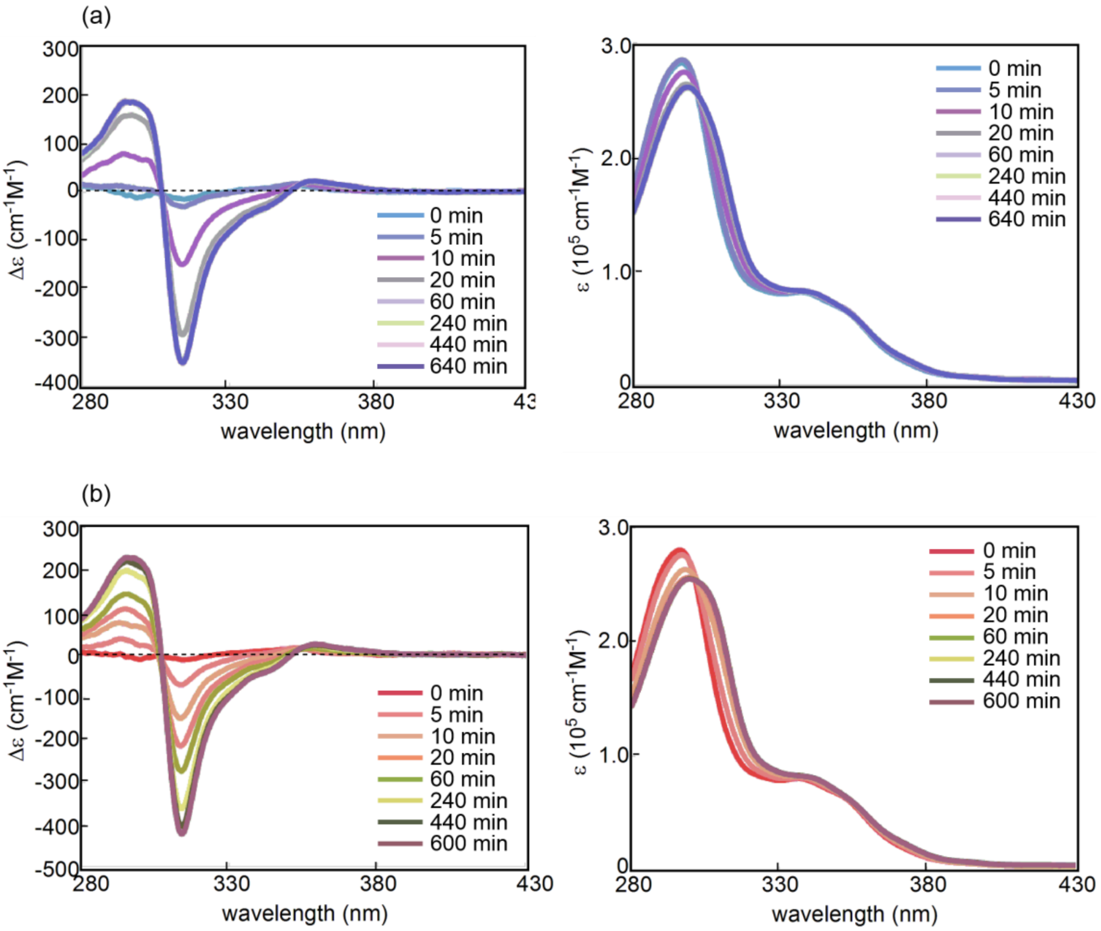

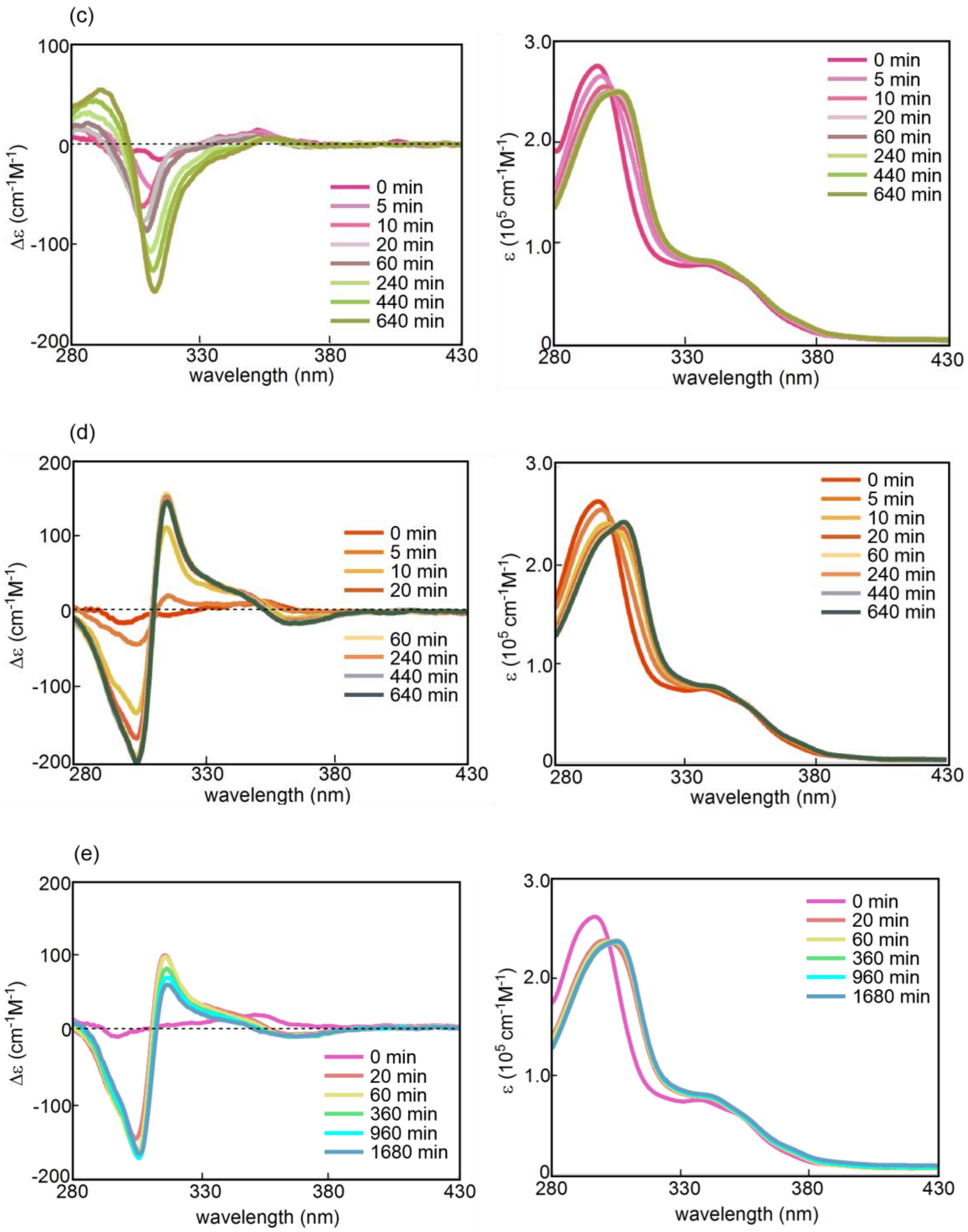

Figure S4. Kinetic analysis under isothermal condition obtained from CD spectra of (*P*)-**1**-C_{16}/(*M*)-**2** in (0.5 mM) at (a) 40 °C, (b) 30 °C, (c) 20 °C, and (d) 5 °C. (e) At 5 °C, long experiment up to 1680 min was also conducted, and selected spectra are shown, which provided Figure 4e.

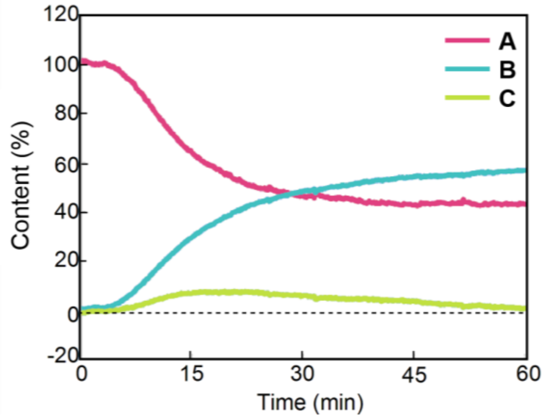

Figure S5. Kinetic analysis at 30 °C under isothermal condition of (*P*)-**1**-C_{16}/(*M*)-**2** in fluorobenzene (0.5 mM) shown by content/time profiles in 60 min by 5 sec intervals, which were obtained from Figures 4a and 4b.

<Isothermal experiment: Effect of concentration>

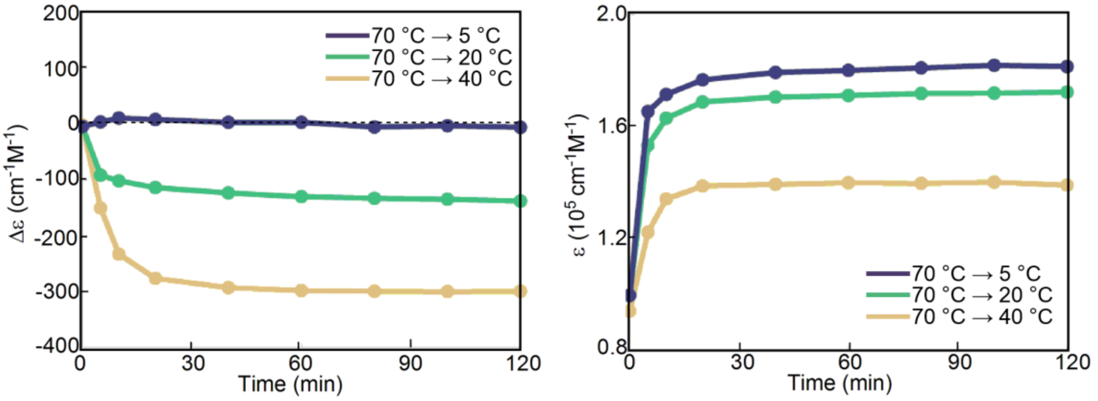

Figure S6. Effect of concentration in kinetic analysis under isothermal condition of 5, 20, and 40 °C shown by Δε (314 nm)/time profiles of (*P*)-**1**-C_{16}/(*M*)-**2** in fluorobenzene (1.0 mM). The lines were drawn between points as a guide for the eyes.

<Figure-eight hysteresis experiments>

A mixture of (*P*)-**1**-C_{16} and (*M*)-**2** in fluorobenzene (0.5 mM) was heated at 70 °C for 10 min, and cooled and heated at the rates and ranges shown in Figures 5, S7, and S8, during which Δε and ε at 314 nm were monitored. Content/temperature profiles are also shown (Figures 5c, S8e and S8f).

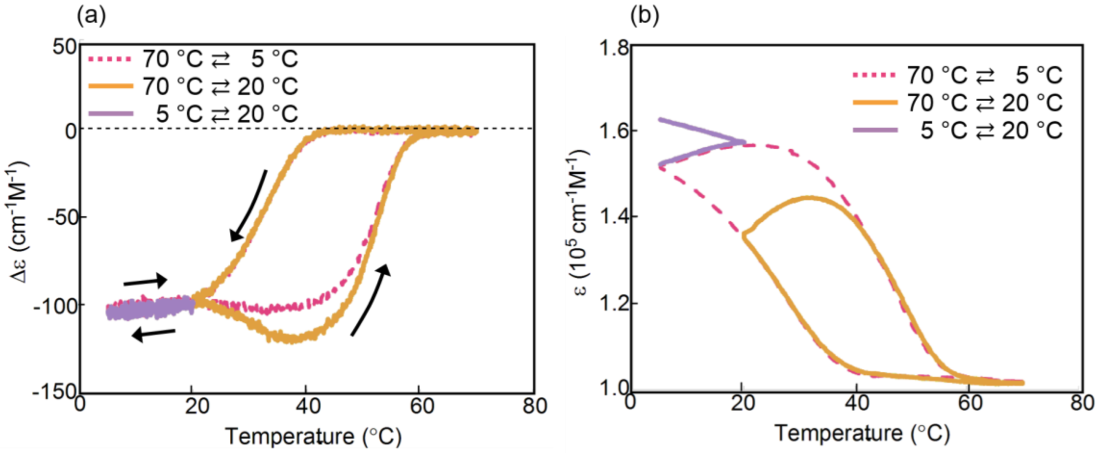

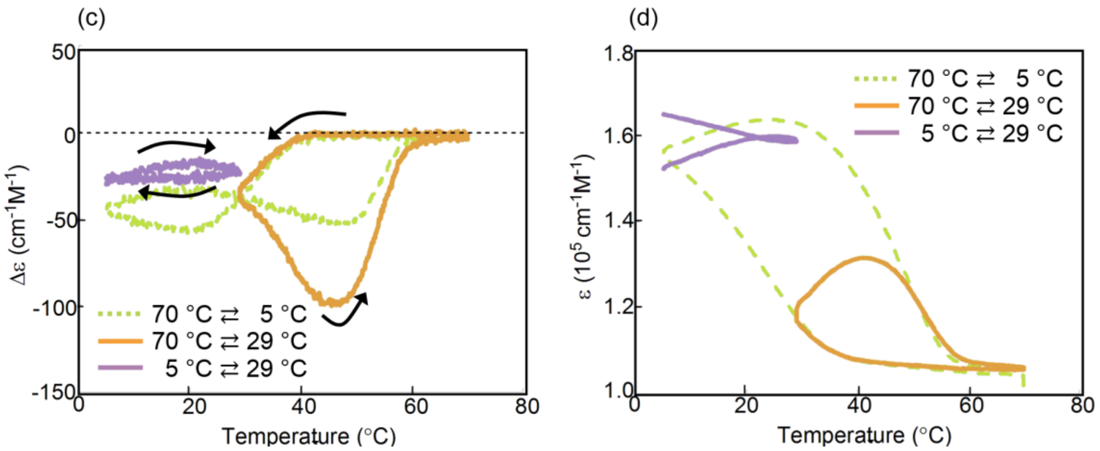

Figure S7. Figure-eight thermal hysteresis in constant-rate cooling and heating experiment of (*P*)-**1**-C_{16}/(*M*)-**2** in fluorobenzene (0.5 mM) shown by (a and c) Δε (314 nm)/temperature profiles and (b and d) ε (314 nm)/temperature profiles at rates of (a and b) 1.5 K/min and (c and d) 2.5 K/min. Temperature was changed (a and b) between 70 and 20 °C and between 20 and 5 °C at the rate of 1.5 K/min and (c and d) between 70 and 29 °C and between 29 and 5 °C at the rate of 2.5 K/min.

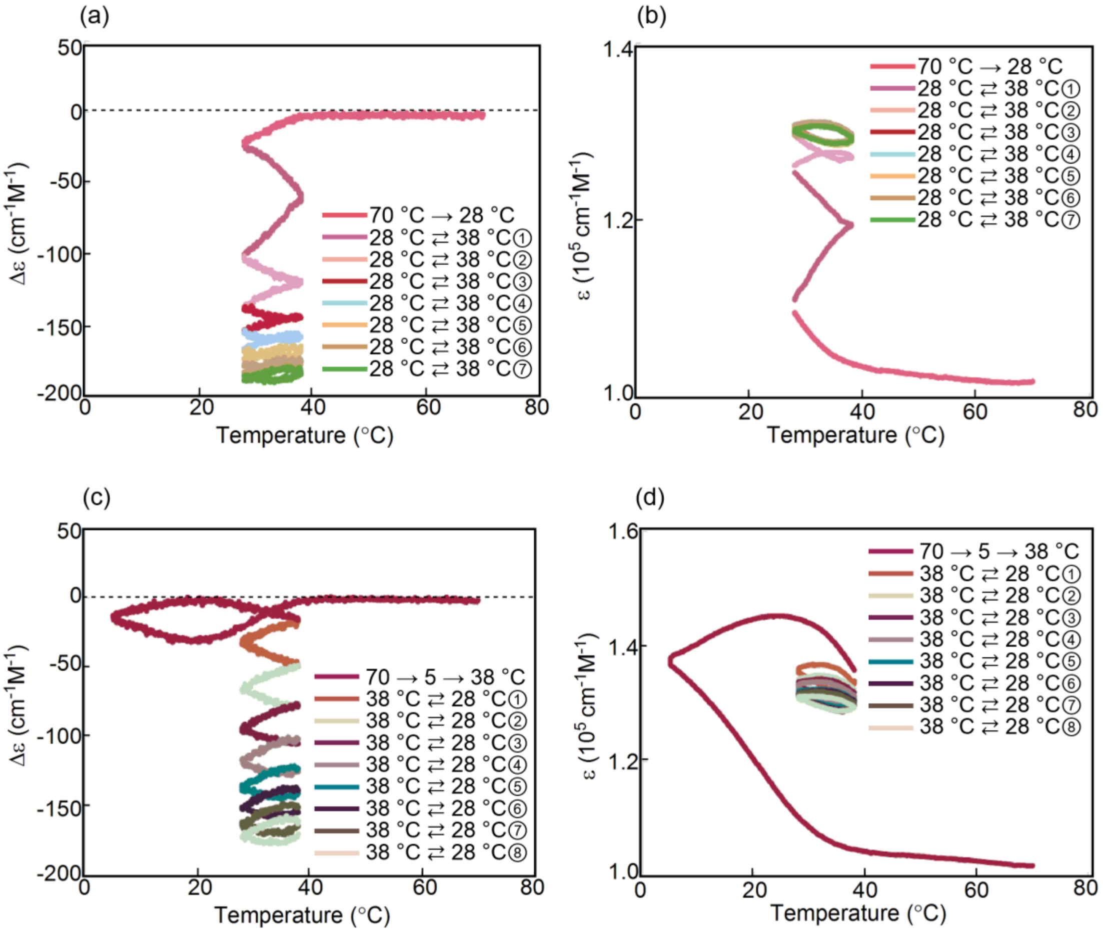

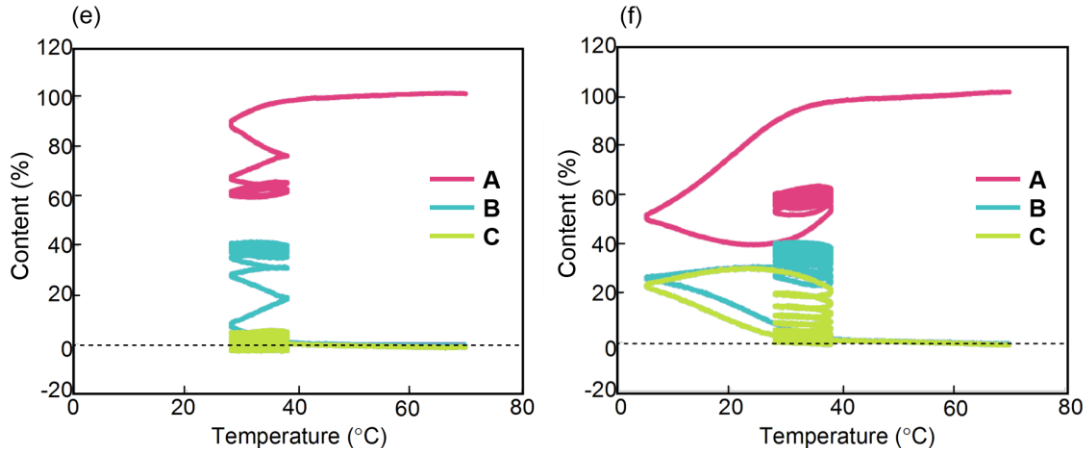

Figure S8. Repeated cooling and heating experiments of (P)-**1**-C_{16}/(M)-**2** in fluorobenzene (0.5 mM) shown by Δε (314 nm)/temperature profiles and ε (314 nm)/temperature profiles at heating/cooling rate of 2.0 K/min. (a and b) Temperature was decreased from 70 °C to 28 °C and then switched between 28 °C and 38 °C. (c and d) Temperature was decreased from 70 °C to 5 °C and then switched between 28 °C and 38 °C. (e and f) Content/temperature profiles at 2.0 K/min are also shown.

3. (*P*)-1/(*M*)-2-C_{16} system

<Formation of hetero-double-helix B>

A mixture of (P)-**1** and (M)-**2**-C_{16} in fluorobenzene (0.5 mM) was heated at 70 °C for 30 min, cooled to 60 °C for 20 min, cooled to 40 °C for 90 min, cooled to 20 °C for 120 min, and cooled to 5 °C for 60 min. CD and UV-vis spectra were obtained at the temperatures (Figure S9).

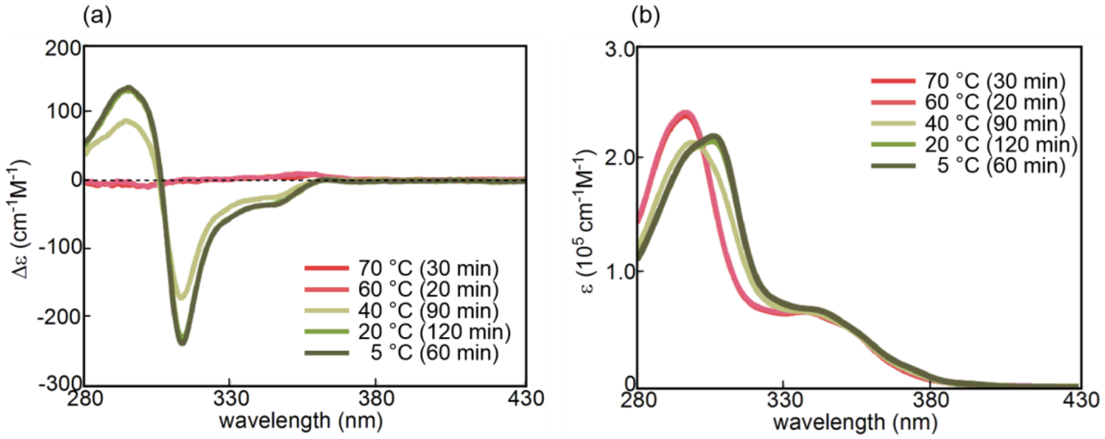

Figure S9. (a) CD and (b) UV-vis spectra of (P)-**1**/(M)-**2**-C_{16} in fluorobenzene (0.5 mM) obtained at 70, 60, 40, 20, and 5 °C.

<Constant-rate cooling and heating experiment>

A mixture of (P)-**1** and (M)-**2**-C_{16} in fluorobenzene (0.5 mM) was heated at 70 °C for 10 min, cooled to 5 °C, and heated to 70 °C at the rates shown in Figures 6a, 6b, and S10, during which Δε and ε at 314 nm were monitored.

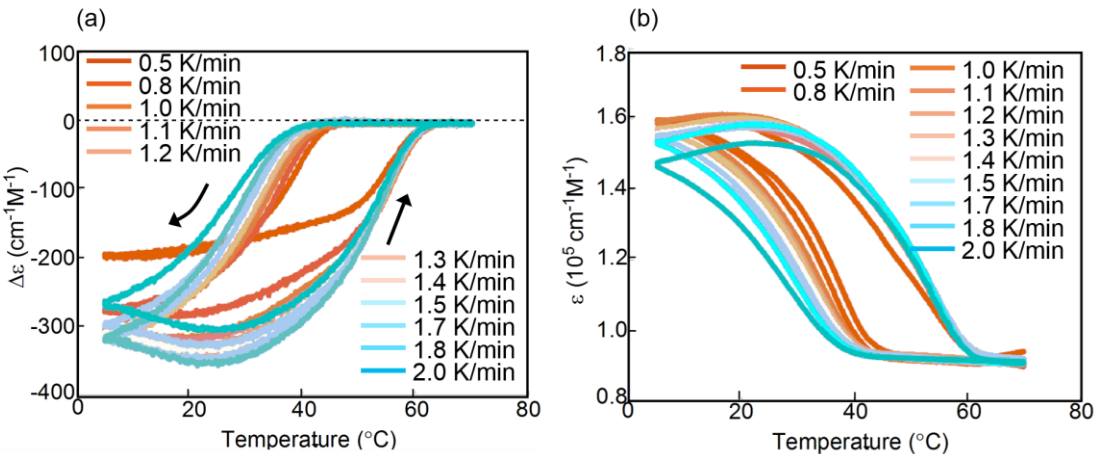

Figure S10. Constant-rate cooling and heating experiments of (*P*)-**1**/(*M*)-**2**-C_{16} shown by (a) Δε (314 nm)/temperature profiles and (b) ε (314 nm)/temperature profiles in fluorobenzene (0.5 mM) at cooling and heating rates between 0.5 and 2.0 K/min. Temperature was decreased from 70 °C to 5 °C and then increased from 5 °C to 70 °C.

<The effect of concentration>

A mixture of (*P*)-**1** and (*M*)-**2**-C_{16} in fluorobenzene at 0.3 mM and 0.7 mM was heated at 70 °C for 10 min, cooled to 5 °C, and heated to 70 °C at the rates shown in Figures S11, during which Δε and ε at 314 nm were monitored.

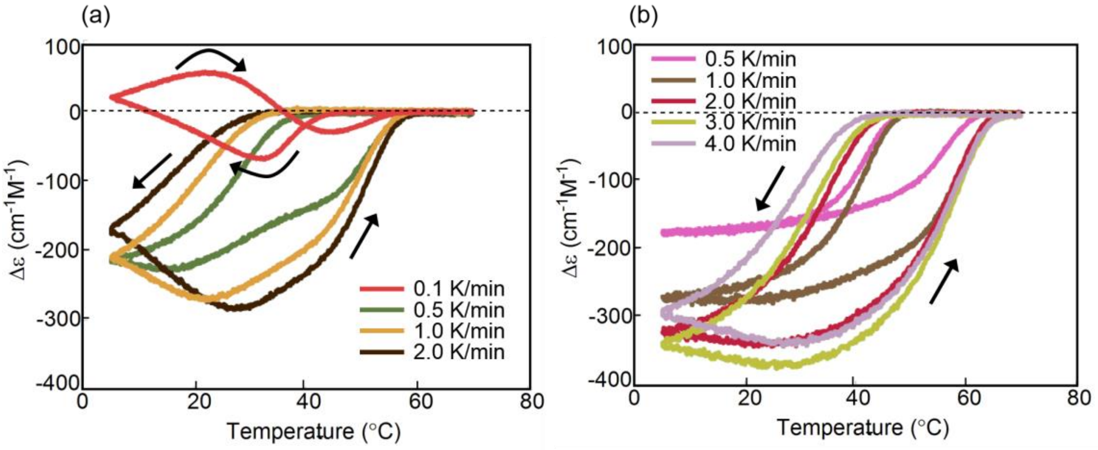

Figure S11. Effect of concentration in constant-rate cooling and heating experiment of (*P*)-**1**/(*M*)-**2**-C_{16} shown by Δε (314 nm)/temperature profiles in fluorobenzene at (a) 0.3 mM and (b) 0.7 mM. Temperature was decreased from 70 °C to 5 °C and then increased from 5 °C to 70 °C.

<Isothermal experiment>

A mixture of (*P*)-**1** and (*M*)-**2**-C_{16} in fluorobenzene (0.5 mM) was heated at 70 °C for 10 min, immediately cooled to 40 and 5 °C. Then, Δε and ε at 314 nm were monitored at the temperatures for 60 min at 5 sec intervals (Figures 7a and 7b). CD spectra were obtained at 40 °C up to 720 min (Figure S12a) and at 5 °C up to 820 min (Figure S12b), from which Figure 7c was obtained.

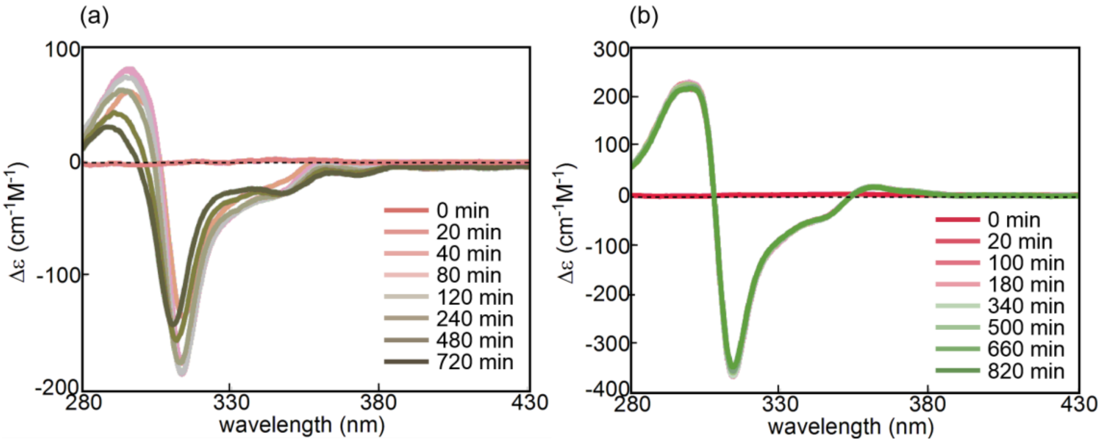

Figure S12. Isothermal experiment of (*P*)-**1**/(*M*)-**2**-C16 at (a) 40 °C for 720 min and (b) 5 °C for 820 min in fluorobenzene (0.5mM) shown by CD spectra. Then, Δε (314 nm)/time profiles in Figure 7c were obtained.

4. (*P*)-1-C16/(*M*)-2-C16 system

<Formation of hetero-double-helix B>

A mixture of (*P*)-**1**-C16 and (*M*)-**2**-C16 in fluorobenzene (0.5 mM) was heated at 70 °C for 20 min, cooled to 60, 40, 20, and 5 °C, and CD and UV-vis spectra were obtained at the temperatures (Figure S13).

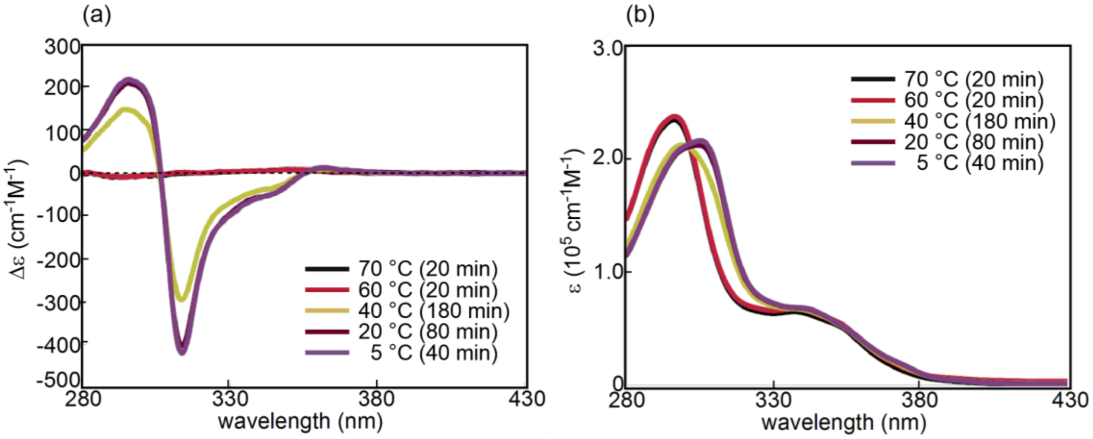

Figure S13. (a) CD and (b) UV-vis spectra of (*P*)-**1**-C16/(*M*)-**2**-C16 in fluorobenzene (0.5 mM) at 70, 60, 40, 20, and 5 °C.

<Constant-rate cooling and heating experiment>

A mixture of (*P*)-**1**-C16 and (*M*)-**2**-C16 in fluorobenzene (0.5 mM) was heated at 70 °C for 10 min, cooled to 5 °C, and heated to 70 °C, during which Δε and ε at 314 nm were monitored (Figures 8a and 8b).

<Isothermal experiment>

A mixture of (*P*)-**1**-C_{16} and (*M*)-**2**-C_{16} in fluorobenzene (0.5 mM) was heated at 70 °C for 10 min, immediately cooled to 40, 30, 20, or 5 °C, and Δε and ε at 314 nm were monitored at the temperatures for 60 min at 5 sec intervals (Figure S14).

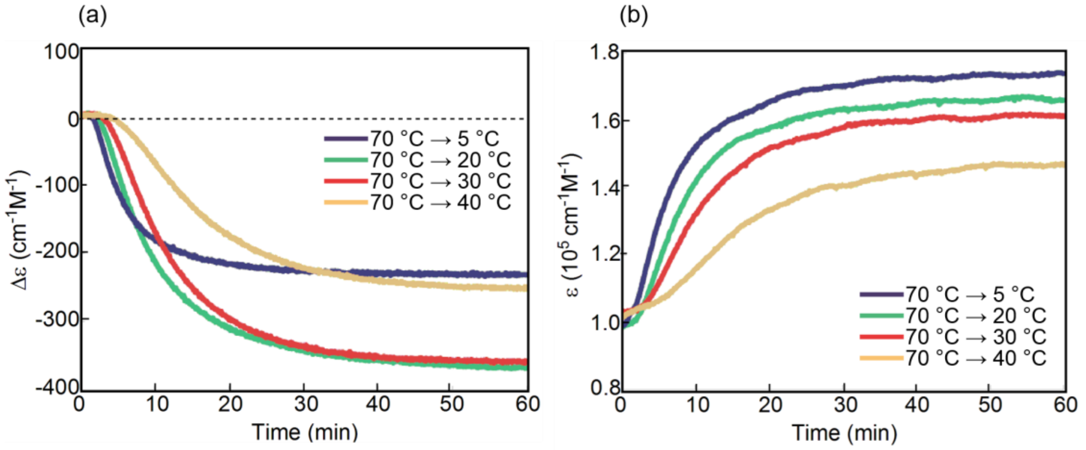

Figure S14. Isothermal experiment of (*P*)-**1**-C_{16}/(*M*)-**2**-C_{16} in fluorobenzene (0.5 mM) shown by (a) Δε (314 nm)/time profiles and (b)ε (314 nm)/time profiles at 40 30, 20, and 5 °C.

5. Comparison of (*P*)-1/(*M*)-2 system[S1]

<Constant-rate cooling and heating experiment>

A mixture of (*P*)-**1** and (*M*)-**2** in fluorobenzene (0.5 mM) was heated at 70 °C for 10 min, cooled to 5 °C, and heated to 70 °C at constant rates between 0.4 and 0.8 K/min, during which Δε and ε at 314 nm were monitored (Figures 9a and 9b).

<Isothermal experiment>

A mixture of (*P*)-**1** and (*M*)-**2** in fluorobenzene (0.5 mM) was heated at 70 °C for 10 min, immediately cooled to 40, 30, 20, or 5 °C, and Δε and ε at 314 nm were monitored at the temperatures for 60 min at 5 sec intervals (Figures 10a and 10b).

6. References

[S1] M. Shigeno, Y. Kushida, M. Yamaguchi, *J. Am. Chem. Soc.*, **2014**, *136*, 7972-7980.
